# Stepwise Development of MAIT Cells in Mouse and Human

**DOI:** 10.1371/journal.pbio.1000054

**Published:** 2009-03-10

**Authors:** Emmanuel Martin, Emmanuel Treiner, Livine Duban, Lucia Guerri, Hélène Laude, Cécile Toly, Virginie Premel, Anne Devys, Ivan C Moura, Florence Tilloy, Stéphane Cherif, Gabriella Vera, Sylvain Latour, Claire Soudais, Olivier Lantz

**Affiliations:** 1 Laboratoire d'Immunologie, Institut Curie, Paris, France; 2 INSERM U932, Institut Curie, Paris, France; 3 Avenir INSERM U925, Faculté de Médecine, Amiens, France; 4 Établissement Français du Sang (EFS), Nantes, France; 5 INSERM, Unité 768, Hôpital Necker-Enfants Malades, Paris, France; Weatherall Institute of Molecular Medicine, United Kingdom

## Abstract

Mucosal-associated invariant T (MAIT) cells display two evolutionarily conserved features: an invariant T cell receptor (TCR)α (iTCRα) chain and restriction by the nonpolymorphic class Ib major histocompatibility complex (MHC) molecule, MHC-related molecule 1 (MR1). MR1 expression on thymus epithelial cells is not necessary for MAIT cell development but their accumulation in the gut requires MR1 expressing B cells and commensal flora. MAIT cell development is poorly known, as these cells have not been found in the thymus so far. Herein, complementary human and mouse experiments using an anti-humanVα7.2 antibody and MAIT cell-specific iTCRα and TCRβ transgenic mice in different genetic backgrounds show that MAIT cell development is a stepwise process, with an intra-thymic selection followed by peripheral expansion. Mouse MAIT cells are selected in an MR1-dependent manner both in fetal thymic organ culture and in double iTCRα and TCRβ transgenic RAG knockout mice. In the latter mice, MAIT cells do not expand in the periphery unless B cells are added back by adoptive transfer, showing that B cells are not required for the initial thymic selection step but for the peripheral accumulation. In humans, contrary to natural killer T (NKT) cells, MAIT cells display a naïve phenotype in the thymus as well as in cord blood where they are in low numbers. After birth, MAIT cells acquire a memory phenotype and expand dramatically, up to 1%–4% of blood T cells. Finally, in contrast with NKT cells, human MAIT cell development is independent of the molecular adaptor SAP. Interestingly, mouse MAIT cells display a naïve phenotype and do not express the ZBTB16 transcription factor, which, in contrast, is expressed by NKT cells and the memory human MAIT cells found in the periphery after birth. In conclusion, MAIT cells are selected by MR1 in the thymus on a non-B non-T hematopoietic cell, and acquire a memory phenotype and expand in the periphery in a process dependent both upon B cells and the bacterial flora. Thus, their development follows a unique pattern at the crossroad of NKT and γδ T cells.

## Author Summary

White blood cells, or lymphocytes, play an important role in defending the body from infection and disease. T lymphocytes come in many varieties with diverse functions. Mucosal-associated invariant T (MAIT) cells constitute a subset of unconventional T lymphocytes, characterized by their invariant T cell receptor (TCR)α chain and their requirement for the nonpolymorphic class Ib (MHC) molecule, MR1. MAIT cells are extremely abundant in human blood and mucosae. Contrary to mainstream T cells, their development requires B cells and commensal microbial flora. To shed light on the little-understood MAIT cells, we used new tools, including an antibody that we recently developed to detect human MAIT cells, and we were able to show that MAIT cell development is a stepwise process, with an intra-thymic selection followed by peripheral expansion. We show that thymic selection is MR1 dependent but requires neither B cells nor the commensal flora, which are both necessary for the expansion in the periphery. In contrast with the other evolutionarily conserved invariant subset, the natural killer T (NKT) cells, we found that MAIT cells exit the thymus as “naïve” cells before becoming antigen-experienced memory cells and expanding in number to represent a significant 1%–4% of peripheral T cells in human blood. In mice, we found that MAIT cells remain naïve and do not expand substantially. We conclude that MAIT cell development follows a unique scheme, where, unlike NKT cells, MAIT cell selection and expansion are uncoupled events that are mediated by distinct cell types in different compartments.

## Abstract 3

Mucosal-associated invariant T cells, the most abundant invariant T cell subset in humans, arise via a distinct developmental pathway that represents a hybrid of that seen for NKT and γδ T cells, two other unconventional T cell subsets.

## Introduction

Unconventional T cells include several T cell receptor (TCR)αβ^+^ major histocompatibility complex (MHC) class Ib restricted, as well as TCRγδ^+^ subsets in both mice and humans [1,2]. They usually display tissue-specific location and are endowed with “natural memory” phenotype and functions, such as the ability to mount rapid responses in the face of a pathogenic challenge. Several pathways of development have been described for these T cell subsets, which show various degree of dependency from the thymus as well as variable requirement for their restricting MHC molecule. At one end of the spectrum, natural killer T (NKT) cells are selected, expand, and acquire their innate-like phenotype and functions in the thymus [3,4]. On the other hand, unconventional (type b) intra-epithelial lymphocytes (IEL) ontogeny can show minimal dependency upon the thymus, as they can escape the thymus at a very early stage and migrate into the gut mucosa where they achieve maturation [5]. They may even develop directly from bone marrow-derived precursors in specific intestinal lymphoid aggregates called cryptopatches [6,7]. Interestingly, both NKT and type b IEL development, phenotype, and functions are seemingly independent from any exogenous antigens such as those derived from the intestinal bacterial flora [8–10]. Finally, it has been recently demonstrated that mouse T10/T22-restricted γδ T cells are able to mature and exit the thymus in the absence of their selecting element, but that thymic selection endows them with a memory phenotype and new effector functions [11].

Among unconventional T cells, only two subsets display both a TCR and selecting MHC class Ib molecules highly conserved between species, the NKT cells and the mucosal-associated invariant T (MAIT) cells (reviewed in [3,4,12]). Indeed, these two populations express highly restricted TCR repertoires consisting of an invariant TCRα chain (mouseVα14/humanVα24-Jα18 for NKT cells; and mVα19/hVα7.2-Jα33 for MAIT cells). Both subsets are selected by hematopoietic cells expressing evolutionarily conserved nonpolymorphic MHC class Ib molecules, encoded in humans in a MHC paralogous region on Chromosome 1: CD1d for NKT cells [13] and MHC-related molecule 1 (MR1) for MAIT cells [14]. NKT cells accumulate in the liver and the spleen, independently of the presence of any exogenous stimuli such as the normal bacterial flora [8], while MAIT cells accumulate primarily in the intestinal lamina propria (LP), in a process dependent on the commensal flora [14]. MAIT cell function is unknown, but the unusual features of these cells and their evolutionary conservation suggest an important role at the interface between innate and adaptive immunity, probably in the intestinal immune system.

For NKT cells, the molecular basis of their specific development begins to be unraveled: contrary to mainstream T cells and γδ T cells, NKT cell development requires the integrity of the SLAM/SAP/fyn pathway with the involvement of homotypic interactions of SLAM receptor family members and CD1d expression on CD4^+^/CD8^+^ (DP) thymocytes [15]. Consequently, NKT cells are absent in mice and humans deficient in the molecular adaptor SAP [16–19]. In addition, mouse and human NKT cells specifically express the ZBTB16 transcription factor very early on during their development and the absence of this factor in luxoid mice leads to a quasi-disappearance of the NKT cells as only a very few number of naive NKT remains [20,21].

Little is currently known about MAIT cell development. They are absent from *nude* mice [22], but MAIT cells are not in sufficiently large number in the thymus for detection by highly sensitive reverse transcription-(RT)-PCR methods in either humans or wild-type (wt) mice ([22] and unpublished data). Previous studies on MAIT cell selection have been based on the measure of the number of these cells in the periphery (in the mesenteric lymph-node [MLN] and in the LP) [14]. MAIT cell development does not require expression of their selecting element on thymus epithelial cells, but the presence of MR1 on B cells is probably necessary for accumulation of MAIT cells in the gut LP [14]. It is therefore unclear whether MR1-dependent selection of MAIT cells really occurs in the thymus, and if so, whether B cells and/or the bacterial flora are required for MAIT cell development in the thymus or peripheral accumulation in the gut.

Herein, we investigated MAIT cell development in both humans and mice. In humans, we used an anti-Vα7.2 monoclonal antibody (mAb) generated in our laboratory to track normal unmanipulated MAIT cells. MAIT cells are rare and display a naïve phenotype both in human thymus and cord blood, while they are abundant and show a homogeneous memory/effector-like phenotype in the blood of all healthy subjects tested. In mice, the over-expression of one of the TCR chains was sufficient to increase the frequency of MAIT cells in central and peripheral lymphoid organs in an MR1-dependent manner. Fetal thymic organ culture (FTOC) and phenotypic studies of the thymus in these mice demonstrated that MAIT cells are selected in the thymus in the absence of B cells and bacterial products. However, most mouse MAIT cells remained naïve in the periphery. The complementary data obtained from humans and mice indicate that MAIT cells are exported from the thymus as naïve cells and subsequently become memory and expand, following interactions with B cells and the commensal flora, in an SAP-independent manner. Thus, MAIT cells use a developmental pathway distinct from both known unconventional T cell subsets and mainstream T cells, showing that they represent a unique subset with its own features.

## Results

### Frequency and Phenotype of Vα7.2 Expressing T Cells in Human Blood

**Figure 1:**
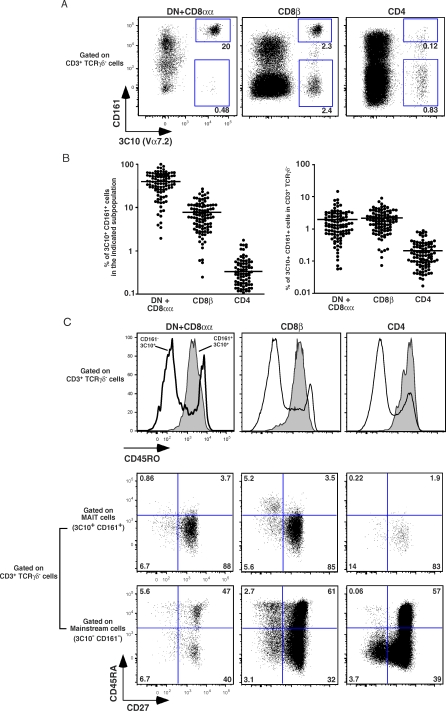
Frequency and Phenotype of Vα7.2 Expressing T Cells in Human Blood (A) Representative 3C10 and CD161 staining gated on αβTCR T cells in DN (+CD8αα), CD8β, and CD4 populations. Percentages of 3C10^+^CD161^+^ and 3C10^+^CD161^−^ are boxed. (B) Percentage of 3C10^+^CD161^+^ cells in indicated DN, CD8β, and CD4 populations (left panel); percentage of DN, CD8β, and CD4 3C10^+^CD161^+^ cells present in the αβTCR T cells (right panel) both estimated by FACS analysis on 104 healthy blood samples. Each dot represents a donor. (C) Representative FACS staining of 3C10^+^CD161^+^ and 3C10^+^CD161^−^ cells in DN, CD8β, and CD4 populations (left, center, and right panels, respectively): expression of CD45RO gated on αβTCR T cells (upper panels) and expression of CD27 versus CD45RA gated on MAIT cells (middle panels) and mainstream T cells (lower panels).

As MAIT cells are defined by the use of an invariant iVα7.2-Jα33 TCRα chain, we developed a monoclonal antibody (3C10) recognizing the TCRα Vα7.2 segment by immunizing Balb/c mice with a recombinant Vα7.2-Jα33/Vβ13 recombinant protein (see Methods). We then used this antibody to measure the number of cells expressing the Vα7.2 segment in blood T cells from healthy donors by assessing the proportion and phenotype of 3C10^+^ cells in the CD4, CD8β and DN subsets of CD3^+^/TCRγδ^neg^ lymphocytes (Figure 1A). Most 3C10^+^ DN (which also includes CD8αα cells) T cells were also found to display strong expression of the CD161 (NKRP1A) marker, and the percentage of 3C10^+^/CD161^hi^ T cells in the DN + CD8αα subset (40.3 ± 21%, m ± SD; range = 1–98%) was very high. In CD8β T cells, the proportion of 3C10^+^/CD161^hi^ cells was variable but significant (7.8 ± 5.5%; 0.2–26.3), while within CD4 T cells, the proportion of 3C10^+^/CD161^hi^ cells was low (0.34 ± 0.34%; 0–1.4), with only a minority of 3C10^+^ T cells also expressing CD161. Reverse transcription (RT)-PCR and polyclonal sequencing of Vα7.2-Cα amplicons obtained from the different FACS-sorted subsets showed that the canonical iVα7.2-Jα33 sequence segregated with the 3C10^+^/CD161^hi^ cells (Figure S1 and unpublished data). The presence of 3C10^+^/CD161^neg^ CD4 and CD8β T cells with no canonical sequence expression demonstrates that 3C10 monoclonal antibody (mAb) is not a clonotypic antibody, but is instead specific for the Vα7 segments, which can be used by mainstream T cells. We were surprised to observe that a significant proportion of CD8α^+^/3C10^+^/CD161^+^ T cells also expressed the CD8β molecule, in conflict with our previous findings [22]. However, a careful analysis showed that the MFI for CD8β in 3C10^+^/CD161^hi^ T cells was only one-quarter to one-sixth that in 3C10^−^/CD161^−^ T cells (Figure S2), suggesting that we missed this subset of cells in our previous FACS-sorting experiments. Interestingly, no such difference in MFI was seen for CD8α (Figure S2), suggesting that at least some of the CD8αβ^+^/3C10^+^/CD161^+^ MAIT cells also express the CD8αα molecule. These results show that MAIT cells can be identified as 3C10^+^/CD161^hi^ T cells, and confirm that they are abundant within the DN and CD8αα^+^ subsets, and scarce in the CD4^+^ T cell subset. However, a large number of these cells may also express an intermediate level of CD8αβ.

The high numbers of DN+CD8αα and CD8βMAIT cells were similar in 104 blood donors, whereas the CD4 subset contained only one-tenth as many such cells (Figure 1B). In adult blood, these cells displayed a homogenous CD45RA^neg^/RO^+^/27^+^/62L^lo^/CCR7^−^/CXCR6^+^ phenotype (Figure 1C and unpublished data) suggesting that blood MAIT cells are memory cells that can migrate into the intestinal tissues.

### MAIT Cells Are Present in the Human Thymus

**Figure 2:**
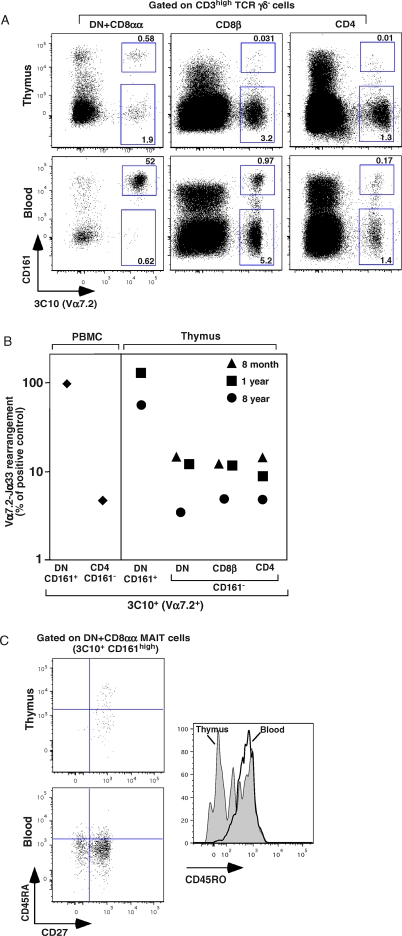
MAIT Cells Are Present in Human Thymus (A) Representative 3C10 and CD161 staining gated on mature CD3^hi^ T cells in DN, CD8β, and CD4 populations in the thymus of a 5-y-old patient, upper panel. The corresponding blood analysis is shown in the lower panel. Percentages are indicated in the quadrants. (B) Quantitative PCR analysis of iVα7.2-Jα33 segment expression normalized to Cα gene expression, on sorted 3C10^+^CD161^+^ DN and 3C10^+^CD161^−^ DN, CD8β, or CD4 populations in the thymus of three different patients (aged 8 months, 1 y, and 8 y). 3C10^+^CD161^+^ DN and 3C10^+^CD161^− ^ CD4 fractions from PBMC were used as positive and negative controls respectively (left panel). The 100% value was defined as that obtained with the 3C10^+^CD161^+^ DN sample from the PBMC, which contains 100 % MAIT cells, whereas the background was defined as the value for 3C10^+^CD161^−^ CD4 cells, which have no canonical sequence. The values for the different samples were normalized with respect to these controls. (C) Representative staining of CD27 versus CD45RA gated on DN MAIT cells in thymus and PBMC (upper and lower left panels, respectively). CD45RO expression by MAIT cells (histogram) in thymus and PBMCs. Representative of three independent experiments.

We used the aforementioned staining strategy to assess the presence of MAIT cells in the human thymus, and observed some 3C10^+^ DN or single-positive CD8β and CD4 T cells (Figure 2A, upper panels). However, by contrast to what was observed with blood (Figure 2A, lower panels), most of the 3C10^+^ cells in the thymus did not express CD161. To assess the identity of these cells, we quantified Vα7.2-Jα33 amplicons in sorted CD161-positive and CD161-negative DN, CD4 and CD8β 3C10^+^ thymocytes, using CD161^neg^/CD4^+^ and CD161^+^/DN blood 3C10^+^ T cells as negative and positive controls, respectively. In two of the three thymuses studied we found a small excess of Jα33 usage (8%–12%) in the CD161^neg^ 3C10^+^ thymocytes (Figure 2B). By contrast, Jα33 was used by 50% to 100 % of the few 3C10^+^/ CD161^hi^ DN thymocytes in the two thymuses containing 3C10^+^/CD161^hi^ T cells. Almost half of this small number of 3C10^+^/CD161^hi^ cells had a naïve (CD45RO^lo^, CD45RA^+^, CD27^+^) phenotype (Figure 2C) ruling out the possibility of blood contamination or recirculating cells. The presence of a few naïve T cells expressing the canonical iVα7.2-Jα33 TCRα chain in the thymus suggests that MAIT cells develop within the human thymus, although this remains to be definitively demonstrated. We therefore used mouse models for the further characterization of MAIT cell ontogeny.

MAIT Cells Ontogeny in Mice Transgenic for the iVα19 TCRα, MAIT Cell Vβ6 TCRβ Chains or Both

Mice have very few MAIT cells and the lack of an antibody specific for the invariant mVα19 TCRα chain precludes the direct analysis of MAIT cells in vivo or ex vivo. We therefore generated mice expressing an invariant mVα19-Jα33 TCRα chain transgene (Tg). A detailed analysis of these mice and their phenotype are provided as supporting information (see Figure S3A and Text S1). As also shown by others [23], the transgenic over-expression of the invariant TCRα chain induces a clear MR1-dependent bias in Vβ chain segment usage towards Vβ6 and Vβ8, reproducing the MAIT cell repertoire of our iVα19 T-T hybridoma [22]. Comparison of Vβ6/Vβ8 bias in the presence or absence of MR1 provided us with an estimate of T cell selection by MR1: at least 50% of the T cells in these mice are MR1 restricted (see Figure S3B, S3C, and Text S1). As shown by others [23] and in the Supporting Information (Figure S3C) MR1-restricted T cells are found in the thymus of iVα19 Cα^−/−^ Tg mice as indicated by a MR1 dependent Vβ6 and/or Vβ8 bias observed in all CD4/CD8/DN T cell subsets.

The forced transgenic expression of a TCRα chain greatly modifies T cell ontogeny [24,25]. We therefore used an alternative strategy based on the transgenic expression of a Vβ6^+^ TCRβ chain from one of the iVα19 T-T hybridoma. As shown previously for mainstream T cells [26] and NKT cells [27], the frequency of the iVα19-Jα33 TCRα chain was increased in these mice in a MR1-dependent manner (see Figure S3D and Text S1).

**Figure 3:**
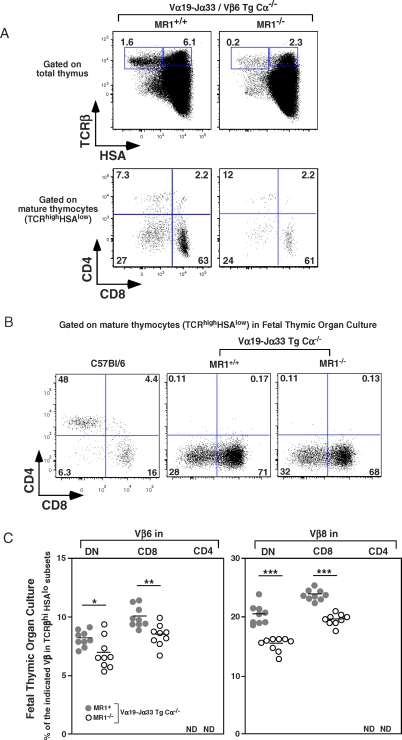
Intra-Thymic Development of MAIT Cells in Mice (A) Representative HSA and TCRβ staining (upper panel) and CD8 and CD4 staining gated on mature thymocytes (lower panel) in iVα19-Jα33/Vβ6 transgenic mice in a MR1^+/+^ Cα^−/−^ or MR1^−/−^ Cα^−/−^ background (left and right panel, respectively). Percentages of mature T cells (TCRβ^hi^HSA^lo^) and intermediate T cells (TCRβ^hi^HSA^hi^) are boxed, (upper panel). Representative of six mice in each group. (B) Representative CD8 and CD4 staining gated on mature T cells in C57Bl/6 and iVα19-Jα33 transgenic mice in a MR1^+/+^ or MR1^−/−^ Cα^−/−^ background in FTOC. (C) Frequency of Vβ6 and Vβ8 expression in mature T cells (TCRβ^hi^ HSA^lo^) in DN, CD8, and CD4 subsets from FTOC of iVα19-Jα33 transgenic Cα^−/−^ mice in the presence (gray circle) or not of (white circle) MR1background. Each dot represents an individual fetal thymus. Representative of two independent experiments. *, *p* = 0.02; **, *p* = 0.002; ***, *p* < 0.0001; ND, not detected.

To further increase the proportion of MAIT cells and to decrease the unwanted TCR specificities due to the endogenous TCRβ chains, we crossed the TCRα Tg and TCRβ Tg mice together in the presence or absence of MR1. The thymuses of the resulting mice were a little smaller than those of wt, in both backgrounds (53 mean [M] ± 26, ± standard deviation [SD], *n =* 15). In the presence of MR1, the mature thymocytes (TCR^hi^/HSA^lo^) were in low numbers in comparison with B6 mice and were mostly DN and CD8 with a concomitant reduction in the CD4 T cell subset (Figure 3A). In the absence of MR1, the frequency and number of mature (TCR^hi^/HSA^lo^) thymocytes decreased by a factor of six to eight, indicating that MR1-restricted MAIT cells are present in the thymus of the MR1^+^ TCRαβ double Tg mice. Importantly, peripheral T cells from these mice strongly up-regulated CD69 expression when cocultured with murine MR1-transfected fibroblasts, confirming that these cells are indeed reactive towards MR1 (S. Huang, EM, S. Kim, L. Yu, CS, et al., unpublished data).

### MAIT Cells Are Found in FTOC

In all the mice studied above, the mature thymocytes were in very small numbers and could be recirculating T cells generated elsewhere. We therefore studied FTOC from iVα19 Tg Cα^−/−^ mice in the presence or not of MR1 to demonstrate formally the intra-thymic development of MAIT cells. Negligible numbers of CD4 T cells were generated whereas the proportion of CD8 and DN T cells was very high among the mature (TCRβ^hi^/HSA^lo^) thymocytes (Figure 3B), similarly to the phenotype found in the adult thymus of mice of the same genetic background although the number of DN cells was lower in adult thymus (Figure S4). Both DN and CD8 T cells displayed a Vβ6 and Vβ8 bias that was dependent on MR1 expression as shown by the significant decrease in Vβ6 (*p <* 0.023 and 0.002 for DN and CD8, respectively) and Vβ8 (*p <* 0.0001) expression in the absence of MR1 (Figure 3C). This MR1 dependent Vβ6/8 usage bias indicates a MR1 dependent selection of many T cells in these FTOC of iVα19 Tg mice. Altogether, these experiments formally demonstrate the existence of an intra-thymic development pathway for MAIT cells.

### Monoclonal MR1-Dependent MAIT Cells Are Found in the Thymus in the Absence of B Cells

**Figure 4:**
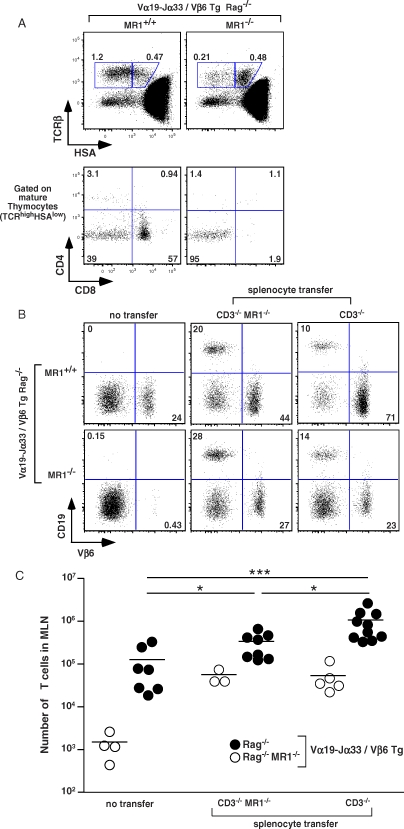
B Cells Are Not Required for Intra-Thymic MAIT Cell Development although B Cells Induce Peripheral Expansion of MAIT Cells (A) Representative HSA and TCRβ staining (upper panel) and CD8 and CD4 staining gated on mature thymocytes (lower panel) in iVα19-Jα33/Vβ6 transgenic mice in a MR1^+/+^ or MR1^−/−^ RAG^−/−^ background (left and right panel, respectively). Percentages of mature and intermediate T cells are boxed (upper panel). Representative of four mice in each group. (B) Representative Vβ6 and CD19 staining gated on MLN lymphocytes from iVα19/Vβ6 double transgenic RAG^−/−^ mice on a MR1^+/+^ (upper panels) or MR1^−/− ^ (lower panels) background without (left panels) or 14 d after MR1^−^ or MR1^+^ CD3ɛ^−/−^ splenocyte transfer. Representative of three to ten mice per group. (C) Absolute numbers of T lymphocytes in the MLN of the different groups analyzed: MR1^+/+^ (filled circles) and MR1^−/−^ (open circles) iVα19/Vβ6 double Tg RAG^−/−^ mice, without or after MR1^−^ or MR1^+^ CD3ɛ^−/−^ splenocyte transfer. *, *p* < 0.021; ***, *p* = 0.0007.

We have previously shown that accumulation of MAIT cells in the periphery depends on the presence of B cells [14]. In contrast with the presence of around 1% B cells in adult thymus, no CD19^+^ cells were detected in the FTOC (unpublished data), suggesting that B cells may not be necessary for thymic MAIT cell selection. To further address the role of B cells in MAIT cell ontogeny, we crossed the iVα19 TCRα and Vβ6 TCRβ Tg lines onto a RAG^−/−^ background, thereby generating mice producing monoclonal MAIT cells in the absence of B cells. In the presence of MR1, the thymus was extremely small (4–6 × 10^5^ cells) with very few mature (TCRβ^hi^/HSA^lo^) cells (Figure 4A, left upper panel), which were either DN or CD8, and only a minimal number of CD4 T cells (Figure 4A, left lower panel). As T cells do not develop at all in the absence of the restricting MHC molecule in mice transgenic for classical TCRs [28,29], this result is compatible with MR1 expression on cells other than B cells. In accordance with this hypothesis, RAG^−/−^ TCRαβ double TCR transgenic mice harbored no mature thymocytes in the absence of MR1 (Figure 4A, right upper and lower panels). Thus, MAIT cells can develop intra-thymically in a MR1-dependent but B cell-independent fashion.

### B Cells Induce Peripheral Expansion of Monoclonal MAIT Cells in Transgenic Mice

The periphery of MR1^−/−^ RAG^−/−^ TCRαβ double Tg mice was almost completely devoid of mature T cells (1.5 ± 0.5 × 10^3^, m ± standard error [SE], *n* = 4) (Figure 4B, upper left panel, and 4C), whereas in the presence of MR1 only a small subset of T cells was found in the MLN (1.3 ± 0.5 × 10^5^, *n* = 7), blood or spleen (Figure 4B upper right panel, 4C, and unpublished data). This suggests that in the absence of B cells, selected MAIT cells are not able to expand and accumulate in the periphery. To directly assess this issue, we performed the following “add-back” experiment: we injected splenocytes devoid of T cells from MR1^+^ or MR1^−/−^ CD3ɛ^−/−^ mice intravenously into MR1^+^ or MR1^−/−^ RAG^−/−^ TCRαβ double Tg mice, and counted the T cells in the MLN after 2 wk. As shown in Figure 4B (lower panel), the splenocytes transfer allowed the same level of reconstitution of B cells in both MR1^+^ and MR1^−/−^ background. The transfer of either MR1− or MR1^+^ B cells into MR1 deficient hosts allowed a clear expansion of the T cells, which however remains in low numbers (5.5 ± 1.3 × 10^4^, *n* = 3 and 5.4 ± 1.8 × 10^4^, *n* = 5, respectively) (Figure 4B, lower middle and right panels, and Figure 4C). This expansion, which was similar with MR1^−^ and MR1^+^ B cells, could either be related to some trophic effect of the B cells or to the modifications of the lymphoid organs they induced. By contrast, when MR1^+^ B cells were transferred into MR1^+^ RAG^−/−^ αβTCR double Tg mice, we found much higher number of T cells (1 ± 0.26 10^6^, *n* = 10) in the periphery (Fig 4B, 4C, and unpublished data). However, MR1 deficient B cell transfer also induced some T cell expansion (3.4 ± 0.8 × 10^5^, *n* = 8) in the periphery of the MR1^+^ TCRαβ double Tg mice. Thus, noncognate interactions (or MR1-independent cognate interactions with membrane molecules exclusively expressed by B cells) between MAIT and B cells might be sufficient to allow MAIT cell expansion. Alternatively, the MR1neg B cells may have captured some MR1 molecules from the MR1^+^ hosts [30] as they are in a “sea” of MR1^+^ cells. However, the much larger number (8-fold with MR1^+^ B cells versus 2.7-fold with MR1^−^ B cells) of MAIT cells found after the transfer of B cells into MR1^+^ RAG^−/−^ αβTCR double Tg mice suggests that cognate interactions are likely involved.

Therefore, in mice where MAIT cells are selected in the thymus but do not expand in the periphery, the adoptive transfer of MR1^+^ B cells is sufficient to promote their accumulation. Interestingly, in all cases MAIT cells acquired a memory (CD44^hi^) phenotype after transfer of B cells (unpublished data). The acquisition of a memory phenotype might be related to a “homeostatic” expansion due to the sudden provision of selecting niches. In any cases, although it could be argued that the splenocyte mixture we injected contains both B cells and other cell types, such as dendritic cells or macrophages, the latter are already present in the host mice before transfer and are obviously not able to induce the peripheral accumulation of MAIT cells. We can therefore formally conclude that B cells are necessary to allow MAIT cells accumulation in the MLN of these monoclonal Tg mice, either through survival, expansion, and/or addressing of MAIT cells to the intestinal territory.

### Human Cord Blood Contains Naive MAIT Cells

**Figure 5:**
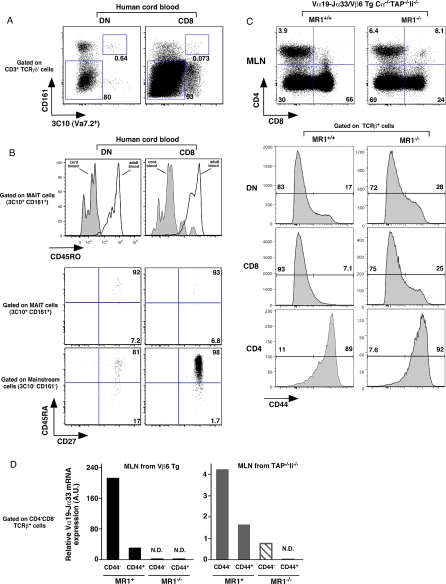
MAIT Cells from Human Cord Blood and Mouse MAIT Cells Display a Naïve Phenotype (A) Representative 3C10 and CD161 staining gated on αβTCR T cells in DN (+CD8αα) and CD8β populations in human cord blood (left and right panel, respectively). The percentages of MAIT cells (3C10^+^CD161^+^) and mainstream T cells (3C10^−^CD161^−^) are boxed. (B) Representative CD45RO expression on DN and CD8β MAIT cells (left and right panels, respectively) in human cord blood and adult blood (upper panel). Expression of CD27 versus CD45RA is gated on MAIT cells (middle panel) or on mainstream T cells (lower panel). (C) Representative CD8 and CD4 staining of MLN from iVα19/Vβ6 double transgenic Cα^−/−^/TAP^−/−^/Ii^−/−^ either on MR1^+/+ ^ (left panels) or MR1^−/−^ (right panels) in upper panel; lower panel expression of CD44 gated on DN, CD8, and CD4 αβTCR T. (D) Quantitative PCR analysis of iVα7.2-Jα33 segment expression normalized to Cα gene expression on sorted CD44^int^ and CD44^hi^ DN cells from the MLN of Vβ6 transgenic mice (left panel) and of TAP^−/−^/Ii^−/−^ mice (right panel) on MR1^+/+^ or MR1^−/−^ background as noted.

The accumulation of murine MAIT cells in the intestinal compartment also requires the presence of the commensal flora [14]. The generation of MAIT cells in FTOC in the absence of exogenous ligand suggests that the commensal flora is necessary not for thymic selection but for the migration and/or peripheral expansion of MAIT cells. We investigated this issue in humans by studying the presence and the phenotype of MAIT cells in immunologically naïve cord blood. The number of CD161^hi^/3C10^+^ MAIT cells was small but measurable in cord blood DN and CD8β T cells (Figure 5A). Polyclonal sequencing of sorted CD161^hi^/3C10^+^ DN cord blood lymphocytes confirmed the presence of the canonical sequence (Figure S6). Strikingly, unlike their adult counterparts, these cord blood MAIT cells displayed a naïve (CD45RA^hi^/CD27^hi^/CD45RO^lo^) phenotype (Figure 5B), making the possibility of a materno-fetal transfusion highly unlikely and demonstrating the presence of naive MAIT cells in human cord blood. Altogether, these results suggest that MAIT cells acquire CD161 expression right before (or concomitantly to) their exit from the thymus, and remain naïve until birth. Colonization with commensal bacteria after birth induces the expansion and/or final maturation of MAIT cells in the periphery.

### Mouse MAIT Cells Display a Naïve Phenotype

We next addressed the question of the naïve/memory status of mouse MAIT cells, which are in much lower number than human MAIT cells. Indeed, in the different Tg mice studied above, the DN and CD8 mature thymocytes are mostly naïve (CD44^lo^/CD122^lo^), while the low numbers of CD4 T cells are constantly CD44^hi^ (Figure S7). As shown and discussed in Figure S5, some of the Vβ6^+^ CD4 T cells found in iVα19 Tg mice are not MR1 restricted but classical MHC class II dependent. To avoid this confounding variable and to increase the proportion of MR1 restricted T cells, we crossed our Tg mice to TAP^−/−^Ii^−/−^ double KO mice, which, being devoid of classical MHC molecules, would select lower number of mainstream T cells. In the absence of classical MHC molecule (Cα^−/−^TAP^−/−^ Ii^−/−^), the MLN T cells found in the iVα19/Vβ6 double Tg mice displayed a naive (CD44^lo^) (Figure 5B) and CD122^lo^ (unpublished data) phenotype. In the absence of MR1, the proportion and the number of CD8 T cells greatly decreased (Figures 5C and S8A) and some increase in CD44 expression was observed with a concomitant increased usage of endogenous Vβ segments (Figure S8B). These results suggest that, in the absence of MR1, pairing of the iVα19 TCRα chain with endogenous TCRβ chains allows selection and peripheral expansion. The proportion and the phenotype of the few CD4 T cells found in these mice remained unchanged in MR1^+^ or MR1^−^ mice indicating that many of these cells were not MR1 dependent.

To further address the question of the naïve/memory phenotype of MAIT cells, we sorted CD44^hi^ and CD44^lo/int^ T cell subsets from the MLN of TAP^−/−^/Ii^−/−^ (to enrich in MAIT cells) and Vβ6 Tg mice on a MR1^+^ or MR1^−/−^ background and quantified the amount of iVα19 transcripts. In both strain of mice, we found that the iVα19 transcripts largely segregated in the CD44^lo/int^ fraction, confirming that wt polyclonal MAIT cells indeed display a naïve phenotype in mice (Figure 5D).

Altogether, these results indicate that murine MAIT cells, like their human counterparts, are selected by MR1 in the thymus without acquiring a memory phenotype. However, they remain naïve in the periphery, even in the presence of B cells and the commensal flora. The difference in number and phenotype of MAIT cells between humans and mice might then be related to the absence of peripheral expansion in the latter.

### Molecular Basis of the Distinct Requirement for MAIT Cells and NKT Cells Development

**Figure 6:**
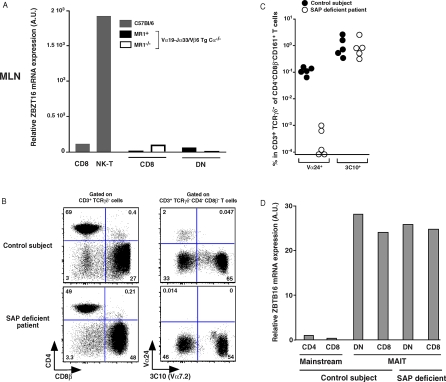
The zbtb16 Transcription Factor Is Absent in Murine MAIT Cells but Expressed in MAIT Cells from Control Subjects and SAP Deficient Patients (A) Quantitative PCR analysis of zbtb16 expression normalized to GAPDH gene expression on sorted CD8 and NKT cells from C57BL/6 mice (left and grey) and from sorted CD8 and DN cells from iVα19-Jα33/Vβ6 transgenic mice in a MR1^+/+^Cα^−/−^ or MR1^−/−^Cα^−/−^ background (right and black and white). (B) Representative CD8β and CD4 staining on gated αβTCR T cells (left panels); 3C10 and CD161 staining gated on DN+CD8αα αβTCR T cells (right panels) from a SAP deficient patient (lower panels) and a control subject (upper panels). (C) Percentage of specific NKT cells (Vα24) and MAIT (3C10) cells in control subjects and SAP deficient patients gated on DNCD161^+^CD3^+^TCRγδ^−^ T cells; each dot represents an individual. (D) Quantitative PCR analysis of zbtb16 expression normalized to GAPDH gene expression on sorted mainstream CD4 and CD8 T cells and DN and CD8 MAIT specific cells from control subject and from sorted DN and CD8 MAIT specific cells from SAP deficient patient.

Contrary to mainstream T cells, NKT cells express the ZBTB16 transcription factor from the first stage of their differentiation in the thymus. In mice deficient for this transcription factor, NKT cells remain naïve, do not expand, and do not colonize the effector organs such as the liver but are found in small numbers in the LN [20,21]. The transgenic forced expression of ZBTB16 controlled by a CD4 promoter induces an effector phenotype in conventional CD4 T cells, indicating that ZBTB16 expression is probably the result of the productive interaction of the iVα14 TCR with CD1d and is responsible for the effector/memory phenotype of NKT cells. The only T cell subset expressing the ZBTB16 transcription factor in addition to the NKT cells was the human MAIT cells [21]. However, we found that mouse MAIT cells did not express ZBTB16 in the periphery (Figure 6A), in accordance with their naïve phenotype. Thus, in contrast with NKT cells, which express ZBTB16 at the early stages of their thymic selection/expansion, ZBTB16 expression in MAIT cells is not related to their selection process. The expression of this transcription factor in MAIT cells is probably linked to an activation step followed by the acquisition of a memory phenotype and peripheral expansion.

Although both NKT cells and MAIT cells are selected on hematopoietic cells, the former subset expands and acquires a memory phenotype within the thymus, whereas MAIT cells seemingly show a more conventional selection process. We sought to investigate the molecular basis for this divergence by studying the SAP-dependency of MAIT cells development. Indeed, CD1d-restricted NKT cells ontogeny involves signaling through the SAP/Fyn/NF-κB signaling pathway, which is triggered by homotypic interactions between SLAM family molecules expressed both on developing NKT cells and selecting cortical thymocytes [15]. We therefore measured the number of MAIT cells in five SAP-deficient patients: whereas no Vα24 NKT cells could be found in these patients, as previously described [16–18,31], MAIT cells were present in normal numbers (Figure 6B and 6C), indicating a probable lack of involvement of the SLAM/SAP/Fyn pathway in MAIT cell development. Interestingly, ZBTB16 was normally expressed by MAIT cells from SAP-deficient patients, confirming that the SLAM/SAP/Fyn pathway is not involved in the induction of this transcription factor expression (Figure 6D). Thus, MAIT cell development clearly diverges from CD1d-restricted NKT cells, despite their common selection on hematopoietic cells [14].

## Discussion

MAIT cells constitute a new subset of MHC class Ib-restricted T lymphocytes conserved among mammals. In this study, we provide for the first time, to our knowledge, phenotypic data on human MAIT cells. We show that they can be tracked by costaining with an anti-Vα7 antibody (3C10) and CD161, allowing us to assess their frequency in the peripheral blood of healthy subjects. Blood MAIT cells are numerous (at least one order of magnitude higher than NKT cells), in accordance with our previous estimates [22]. As previously published, a majority of MAIT cells display a CD4^−^CD8^−^ (DN) or CD8αα phenotype, but few of them are CD4^+^. We now show that MAIT cells can express intermediate levels of CD8αβ, which were missed in our first characterization. The low level of CD8αβ expression may allow developing CD8αβ^+^ MAIT cells to escape negative selection. These data imply that selection of MAIT cells is independent of coreceptors, but that an Ag-driven process induces differential expansion of the different subsets, as also suggested by the differences in Vβ usage between DN/CD8 and CD4 T cells in the MLN and LP of iVα19 TCRα Tg mice.

Two other groups have generated iVα19 TCRα Tg mice [23,32,33]: in only one of these studies [23], the comparison was made between MR1^+^ and MR1-deficient backgrounds allowing the distinction between selection-driven features and those related to the transgenic artifact. Our results are similar in terms of organ cellularity, CD4/CD8/DN subset distribution, and Vβ6/8 bias. The careful study of the phenotype of our mice in the different background allowed us to show that MAIT cells are DN and CD8 and not much CD4. We also observed no significant expression of NK1.1, CD25, CD69, or ICOS on the T cells of iVα19 or Vβ6 single or double Tg mice (unpublished data). Moreover, we studied the phenotype of wt murine MAIT cells and found that NK1.1 is expressed on <30%–50 % of MAIT cells from the LP of wt C57Bl/6 mice (unpublished data). Most significantly, unmanipulated human MAIT cells express none of these markers, given that CD161 (NKR-P1A) is not the human ortholog of NK1.1 (NKR-P1B/C in B6 mice). The differences observed between our mice and those studied in previous investigations are probably due to experimental procedures, such as differences in the transgenic vectors used or to differences in the commensal flora of different animal facilities as MAIT cells require commensal flora to expand [14]. In any case, we believe that these markers, including NK1.1 in particular, cannot be used to reliably track MAIT cells in vivo.

We have previously shown that MAIT cells accumulate in the MLN and the gut LP. However, MAIT do not accumulate in sufficient numbers in the thymus of wt mice to be detectable by sensitive reverse transcription (RT)-PCR methods, precluding the possibility to discriminate between intra-thymic selection per se and peripheral expansion. Moreover, it has been suggested recently that the development of some unconventional intra-epithelial lymphocyte (IEL) T cell populations requires a functional thymus but actually takes place in the gut mucosa [5,7], while T10/T22-restricted γδ T cells can mature and migrate in the periphery in the absence of thymic selection [11]. Herein, we demonstrate for the first time, to our knowledge, that MAIT cells are indeed selected in the thymus in an MR1-dependent manner, but that B cells and bacteria are not required for their initial intra-thymic generation, whereas both are necessary in the periphery for MAIT cell expansion. Therefore, MAIT use a unique stepwise developmental pathway that is distinct from both NKT and conventional T cells.

Our data clearly demonstrate that B cells are not the selecting cells in the thymus, but are necessary for the accumulation of MAIT cells in the MLN and LP, by promoting either expansion, survival, and/or addressing to the gut compartment. We have previously shown that MAIT cells are selected on non-T hematopoietic cells [14]; therefore our data implicate most probably myeloid cells such as macrophages or dendritic cells in intra-thymic MAIT cell selection.

Recent data from Bendelac and coworkers and from our own lab show that MAIT cells and NKT cells exclusively share the expression of the transcription factor PZLF (ZBTB16) [21], emphasizing a close lineage relationship between these two distinct subsets. However, the data presented in this paper nonetheless describe profound differences during the ontogeny of these two populations. We clearly show that MAIT cell selection is not accompanied by activation (as is the case with NKT cells [34,35]), since it does not lead to local expansion and acquisition of a memory phenotype. Accordingly, ZBTB16 was not expressed by mouse MAIT cells (Figure 6A), which display a naïve phenotype while human MAIT cells acquired ZBTB16 together with a memory phenotype after birth. It has been also shown recently that the ontogeny of NKT cells is heavily dependent upon homotypic interactions between SLAM family members and the SAP signaling pathway [15]. We show here that MAIT cells development is apparently unaffected by SAP mutations in humans, and this might be related to the fact that postselection MAIT cells display a naïve phenotype in both humans (Figure 2C) and mice (Figure 5C). In agreement with the mouse data showing that SAP deficiency does not prevent the expression of the ZBTB16 molecule at the early stages of NKT ontogeny, ZBTB16 expression in MAIT cells was unaffected in SAP deficient patients.

The absence of ZBTB16 expression by mouse MAIT cells could be related to the cleanness of the animal facilities, which would not provide the ligand or inflammatory context necessary for MAIT cell expansion. Alternatively, one key genetic component may be missing in mice because of the genetic bottleneck that laboratory mouse strains have been through without selection pressure by the putative function mediated by the MAIT cells. Finally, how ZBTB16 expression is acquired in the thymus by NKT cells and in the periphery by human MAIT cells is an open question. It is probably related to the context of the interactions between the iTCR and the selecting element.

The acquisition of a memory phenotype by human MAIT cells after birth is most likely linked to the colonization of the gut (and other mucosae) with the commensal flora. We speculate that B cells provide the link between the gut bacterial flora and MAIT cells. Direct or indirect (via epithelial cells or dendritic cells [36,37]) interactions between B cells and bacteria in the gut could either induce MR1 expression and/or endow B cells with specific costimulatory properties, akin to the SLAM-mediated costimulation of developing NKT cells. However, the absence of ZBTB16 expression, and the concomitant naïve phenotype of mouse MAIT cells implicate that some other signals are required for their full innate-like differentiation. The dependency upon bacteria of MAIT cell expansion provides another example of the strong mutualism between the commensal flora and the development of the mammalian immune system [38].

In conclusion, we show here that besides their similarities, MAIT cell ontogeny is clearly different from NKT cells. Postselection NKT thymocytes already display innate-like properties, with a high frequency and a memory phenotype, whereas postselection MAIT cells are still naïve and need contacts with both B cells and bacteria to expand, acquire a memory phenotype in human, and accumulate in the gut. In this respect, they may be compared to Vδ2^+^ T cells, which appear in blood as naïve but become memory soon after birth [39,40]. The striking high frequency of MAIT cells in the blood of healthy subjects suggest that they play a prominent role in various diseases, either of infectious, tumoral, or auto-immune origin.

## Methods

### Mice*.*

CD3ɛ-deficient B6 mice, Cα deficient mice (N9 to C57Bl/6 [B6]) and Ii^−/−^ (B6/129) mice were obtained from the CNRS CDTA central animal facility (Orléans, France). TAP^−/−^ mice in a B6/129 background were obtained from the Jackson laboratory. Cα^−/−^, Ii^−/−^, and TAP^−/−^ mice were intercrossed to obtain double and triple deficient mice. MR1^−/−^ mice have been described elsewhere [14]. MR1^−/−^ mice, kindly provided by S. Gilfillan, were backcrossed for more than ten generations onto B6 mice. They were also crossed to TAP^−/−^/Ii^−/−^ mice. All the TAP^−/−^/Ii^−/−^ mice had a mixed B6/129 background. iVα19-Jα33 and Vβ6 transgenic mice were obtained as previously described [41] by cloning PCR products obtained from a Vα19/Vβ6 T-T hybridoma cDNA into the TCR alpha or beta shuttle vector [26]. The TCRα and TCRβ constructs were injected into B6/DBA2 F1 and B6 eggs, respectively, at the CNRS transgenic facility (C. Goujet, Villejuif, France). Three iVα19 TCRα founder lines were backcrossed for at least ten generations onto Cα^−/−^ B6 mice before further crossing. Most of the results were obtained with two founder lines expressing six and eight copies of the transgene. At this stage, these two founder lines were also crossed with the Vβ6 TCRβ Tg lines. They were also crossed with the RAG2^−/−^ CD45.1 B6 line and then with the Vβ6 TCRβ Tg B6 lines to obtain iVα19/Vβ6 RAG^−/−^ CD45.1 B6 lines. The CD45.1 allotype marker was also introduced into one of the iVα19 Cα^−/−^ B6 lines. The two TCRα Tg lines were also crossed with Cα^−/−^/TAP^−/−^/Ii^−/−^. The MR1-deficient allele in a B6 background was introduced into all lines. All mice were housed in our SPF colony and genotyped by PCR or FACS staining, as appropriate. Live animal experiments were done in accordance with the guidelines of the French Veterinary Department.

### 3C10 anti-TCRα Vα7.2 segment monoclonal antibody generation.

The canonical TCRα chain and the associated Vβ13 TCRβ chains were cloned (ET and OL), from an iVα7.2-Jα33 T cell clone (J.F. Davodeau, ET, M. Bonneville, and OL, unpublished data). Using this template, L. Teyton (SCRI) generated a recombinant heterodimeric protein, which was used to immunize Balb/c mice. After fusion with SP2/0, hybridoma specifically staining a high proportion of CD3^+^ DN and a proportion of CD8α but few CD4 T cells were selected and cloned. The 3C10 hybridoma was chosen for further characterization because, according to quantitative PCR, the Vα7.2-Jα33 TCRα chain segregated with the 3C10 positive fraction in DN T cells. When transfectants expressing different mouse TCRα and TCRβ chains and a chimeric human iVα7.2-mouse Cα were stained with the 3C10 supernatant, only transfectants harboring the Vα7.2 segment displayed positive staining demonstrating that 3C10 was not an anti-TCRβ chain antibody (unpublished data). Quantitative PCR analysis of the 3C10^+^ and 3C10^−^ fractions using primers for all Vα segments, demonstrated the absence of significant cross-reactivity between the 3C10 mAb and other Vα segments (unpublished data). The 3C10 antibody was biotinylated and detected with streptavidin PE-Cy7 (BD Pharmingen).

### Cell preparation.

Cell suspensions were prepared from thymus, spleen, peripheral or mesenteric lymph nodes, and gut LPL as previously described [14].

### Flow cytometry*.*

Flow cytometry was performed with directly conjugated antibodies (BD Pharmingen) according to standard techniques with analysis on FACS Aria and LSRII flow cytometers (Becton Dickinson). DAPI and a 405-nm excitation were used to exclude dead cells. The following antibodies, mostly from BD Pharmingen or eBiosciences, were used in mice: anti-CD45.2-Fitc (104), anti-CD3ɛ-PC7 (145–2C11), anti-CD5-APC (Ly-1), anti-Vβ6-PE (RR4–7), anti-CD44-PE or APC (IM-7), anti-CD45.1-PE (A20), anti-βTCR-PC5 (H57–597), anti-CD19-PE, Fitc or APC-Cy7 (1D3), anti-CD8α-APC-Cy7 (Ly-2), and anti-CD4-PE-Texas Red (L3T4).

For human stainings, the following were used: anti-CD4-APC-Cy7 (RP4-T4), anti-CD3ɛ-Alexa 700 (UCHT1), anti-TCRγδ-PC5 (IMMU510), anti-CD8β-PE Texas Red (2ST8.5H7), anti-CD45RO-Fitc (UCHL1), anti-CD45RA-PE (HI100), anti-CD27-Fitc (M-T271), anti-CCR7-PE (150503), anti-CD62L-PE (Dreg 56), anti-CXCR6-PE (56811), anti-CD161-APC (DX12), and anti-CD8α-PE Alexa700 (RPA-T8).

### Human samples.

Fragments of human thymuses were operating residues from children undergoing cardiac surgery. Thymuses were cut into small pieces in cold 0.5% BSA PBS. The resulting cell suspension was centrifuged to exclude aggregates. The supernatant was recovered and the cells were washed again with 0.5% BSA PBS. Thymocytes were then counted and labeled.

Blood samples were obtained from healthy donors from the blood bank in accordance with institutional regulations.

### Adoptive transfer.

Spleens from CD3ɛ^−/−^ MR1^+^ mice were harvested and 10^7^ splenocytes were IV injected into MR1^+^ or MR1^−^ TCRαβ double Tg Rag^−/−^ mice. 2 wk later, the blood, spleen, and MLN were harvested and analyzed by FACS.

### FTOC.

iVα19 Tg Cα^−/−^ MR1^−/−^ or MR1^+/+^ female mice were caged one night with the respective genotype male mice. Pregnant female mice were sacrificed at day 14. Uterine corns were harvested in CO2-independent medium supplemented with 5% FCS and penicillin/streptomycin (Invitrogen). Fetal thymuses were harvested and cultured in 300 μl of IMDM medium supplemented with 10% FCS, 50 μM beta-mercaptoethanol, 10 mM Hepes, 1 mM Sodium pyruvate, 2 mM L-glutamine, and penicillin/streptomycin in transwell plates from Costar (0.4 μm, 12 wells). The medium was changed every 3 d over a period of 6–8 d.

### Amplification, cloning, and sequencing of Vα19-Jα33 junctions and Vβ6 segments.

Molecular biology methods: RNA extraction, reverse transcription, TCR primers, PCR methods, and quantitative polyclonal PCR and polyclonal sequencing were carried out as previously described [14,22].

## Supporting Information

Figure S1 The Canonical Sequence of MAIT Cells Is Found only in the 3C10^+^CD161^+^ Population Sequence of Vα7.2-Cα amplicons obtained after polyclonal sequencing, with a specific Vα7.2 primer, of αβTCR T cells sorted from either 3C10^+^CD161^high^ or 3C10^+^CD161^lo^ cells in DN, CD8β, and CD4 subsets. Representative of four independent experiments.
(866 KB PDF)

Figure S2 CD8β Mean Fluorescence Intensity on MAIT Cells Representative staining of MAIT cells (3C10^+^CD161^high^) and mainstream T cells (3C10^−^CD161^lo^) gated on CD4^−^ αβTCR T cells in PBMCs (upper panel). Representative CD8α/CD8β staining gated on mainstream CD4^−^ T cell and CD4^−^ MAIT cells (left and right middle panels, respectively). CD8α and CD8β expression by CD4^−^ mainstream T cells or CD4^−^ MAIT cells (left and right lower panels, respectively). Percentages of mainstream T and MAIT cells are boxed, upper panel. Representative of three independent experiments.
(98 KB PDF)

Figure S3 Phenotype of iVα19-Jα33 and Vβ6 Transgenic Mice (A) Absolute number of mature T cells in lymphoid organs of C57Bl/6 (black square) and iVα19-Jα33 transgenic mice in a Cα^−/−^ (gray circle) or Cα^−/−^TAP^−/−^Ii^−/−^ (black circle) background. Mononuclear cells from thymus (a), spleen (b), peripheral lymph nodes (PLN) (c), MLN (d), and gut LP (e) were harvested as described, counted, and the percentage of TCRβ^+^ cells was determined by FACS analysis. Each dot represents an individual mouse.
(B) Frequency of Vβ6 (left panel) and Vβ8 (right panel) in DN, CD8, and CD4 populations, analyzed by FACS analysis, in αβTCR T cells in the MLN (upper panel) and LP (lower panel) of C57Bl/6 and iVα19-Jα33 transgenic mice in a Cα^−/−^ or Cα^−/−^TAP^−/−^Ii^−/−^ background.
(C) Vβ6 (left panel) and Vβ8 (right panel) bias in transgenic mice in DN, CD8, and CD4 populations on different backgrounds, analyzed by FACS, in mature thymocytes of C57Bl/6 and iVα19-Jα33 transgenic mice on a Cα^−/−^ or Cα^−/−^MR1^−/−^ (white circle) background. Each dot represents an individual mouse.
(D) Quantitative PCR analysis of iVα19-Jα33 segment expression normalized with respect to Cα gene expression on sorted DN, CD8, or CD4 T cells from thymocytes or MLN lymphocytes of MR1^+^ or MR1^−/−^ C57Bl/6 mice. Each dot represents an individual mouse.
(149 KB PDF)

Figure S4 Bias in the Coreceptor Usage in the Thymus of iVα19-Jα33 Transgenic Mice Representative CD8/CD4 staining gated on total thymus (upper panels) and on mature thymocytes (TCRβhigh HSAlow) (lower panels) of C57Bl/6 and iVα19-Jα33 transgenic mice on a MR1^+/+^ or MR1^−/−^ Cα^−/−^ background either (left, middle, and right panels, respectively). percentages of CD4, CD8, and DN T cells populations are boxed. Representative of ten independent experiments.
(71 KB PDF)

Figure S5 Vβ6 and Vb8 Bias in Transgenic Mice on Different Backgrounds Frequency of Vβ6 (left panel) and Vβ8 (right panel) in DN, CD8, and CD4 populations, analyzed by FACS, in αβTCR T cells from the MLN of C57Bl/6 (black square) and iVα19-Jα33 transgenic mice on a Cα^−/−^ (gray circle) or Cα^−/−^MR1^−/−^ (white circle) background; upper panels. Frequency of Vβ6 (left panel) and Vβ8 (right panel) in DN, CD8, and CD4 populations, analyzed by FACS, in αβTCR T cells from the MLN of C57Bl/6 (black square) and Cα^−/−^TAP^−/−^Ii^−/−^ (black circle) or Cα^−/−^TAP^−/−^Ii^−/−^MR1^−/−^ (white circle) background; lower panels. Each dot represents an individual mouse.
(76 KB PDF)

Figure S6 The Canonical Sequence of MAIT Cells Is Found on the 3C10+CD161high DN Human Cord Blood T Cells Sequence of Vα7.2-Jα33 amplicons obtained after polyclonal sequencing, with a specific Vα7.2 primer, of 3C10^+^CD161^hi^ DN and 3C10^+^CD161^lo^ CD8 αβTCR T cells sorted from cord blood. Representative of two separate experiments.
(229 KB PDF)

Figure S7 MAIT Cells Display a Naive Phenotype Representative staining of CD44 expression on DN, CD8, and CD4 gated on mature T cells (TCR^hi^HSA^lo^) from the thymus left part; and from TCR^hi^ T cells from MLN right part, in C57BL/6 and Vβ6, iVα7.2-Jα33/ Cα^−/−^ and in iVα19-Jα33/Vβ6/ Cα^−/−^ transgenic mice. Shaded grey histogram is on an MR1^+/+^ background while black line is on an MR1^−/−^ background.
(323 KB PDF)

Figure S8 The Absence of Classical MHC Molecules Show a Dramatic MR1 Dependant CD8 Profile of MAIT Cells (A) Absolute numbers of mature T cells from the thymus and the MLN of iVα19-Jα33/Vβ6/Cα^−/−^/TAP^−/−^/Ii^−/−^ transgenic mice on a MR1^+/+^ (black diamond) or MR1^−/−^ (white diamond) background.
(B) Representative CD44 and a pool of endogenVβ staining of DN, CD8, and CD4 gated on TCRβ^hi^ T cells from MLN of C57BL/6 (upper panel) and iVα19-Jα33/Vβ6/ Cα^−/−^/TAP^−/−^ /Ii^−/−^ transgenic mice on a MR1^+/+^ (middle panel) or MR1^−/−^ (lower panel) background.
(188 KB PDF)

Text S1 Description and Discussion of the iVα19 TCRα Chain and Vβ6 TCRβ Chain Single Transgenic Mice (68 KB DOC)

## Abbreviations

FTOC: fetal thymic organ culture
LP: lamina propria
MAIT: mucosal-associated invariant T
MHC: major histocompatibility complex
MLN: mesenteric lymph node
MR1: MHC-related molecule 1
NKT: natural killer T
TCR: T cell receptor
wt: wild type
