# Supplementary material for: Stepwise Development of MAIT Cells in Mouse and Human
Source: PLoS Biol. 2009 Mar 10;7(3):e1000054. doi: 10.1371/journal.pbio.1000054 (PMC2653554; doi:10.1371/journal.pbio.1000054)
Supplement: Supplementary file 1 [file pbio.1000054.sg001.pdf]

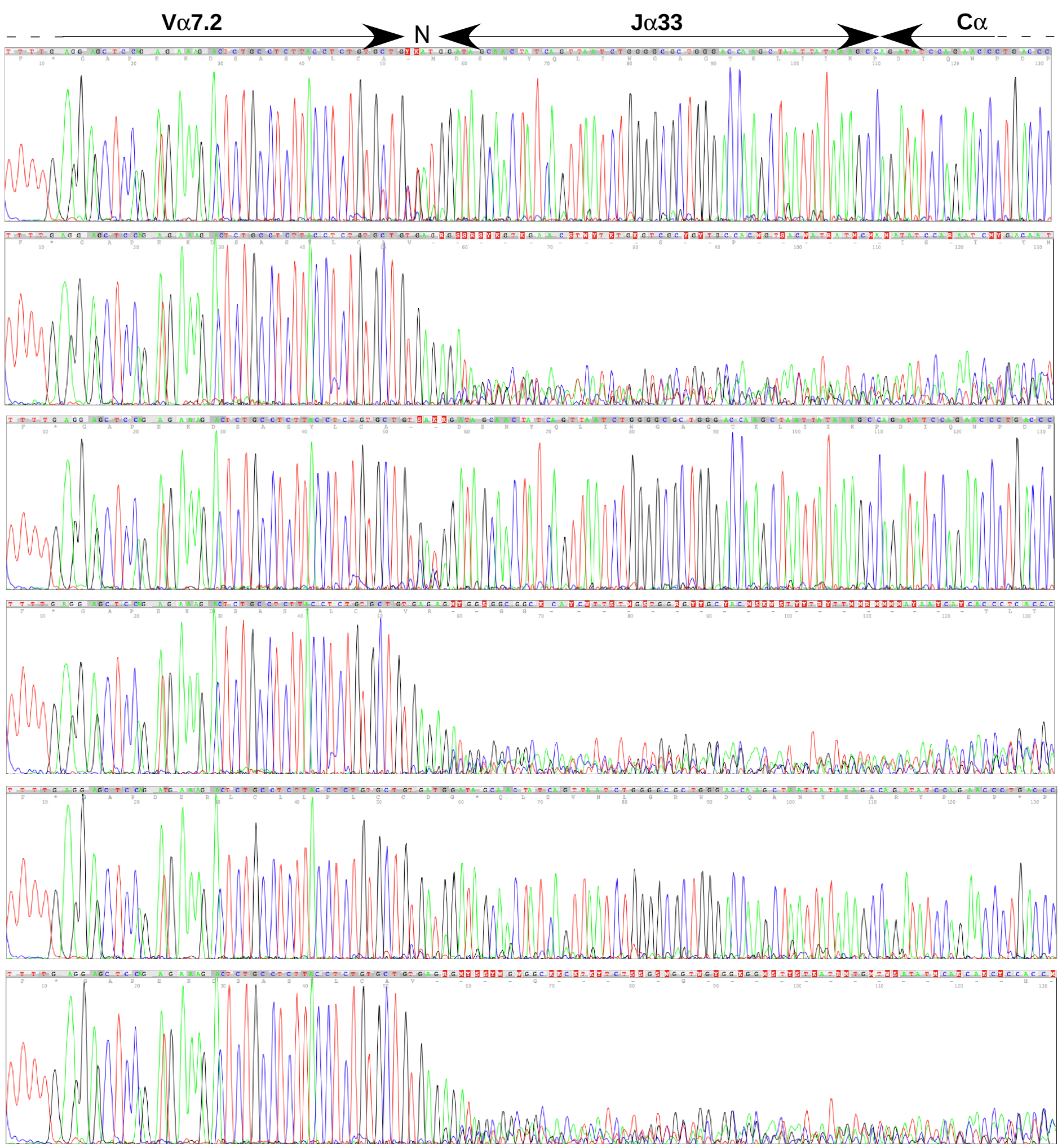

$CD3^{+}$ $TCR\gamma\delta^{-}$ cells fractions sorted	Canonical sequence
DN + CD8αα $3C10^{+}CD161^{high}$	+
DN + CD8αα $3C10^{+}CD161^{low}$	-
$CD8\beta^{+}$ $3C10^{+}CD161^{high}$	+
$CD8\beta^{+}$ $3C10^{+}CD161^{low}$	-
$CD4^{+}$ $3C10^{+}CD161^{high}$	+
$CD4^{+}$ $3C10^{+}CD161^{low}$	-
