# Supplementary material for: Stepwise Development of MAIT Cells in Mouse and Human
Source: PLoS Biol. 2009 Mar 10;7(3):e1000054. doi: 10.1371/journal.pbio.1000054 (PMC2653554; doi:10.1371/journal.pbio.1000054)
Supplement: Supplementary file 2 [file pbio.1000054.sg002.pdf]

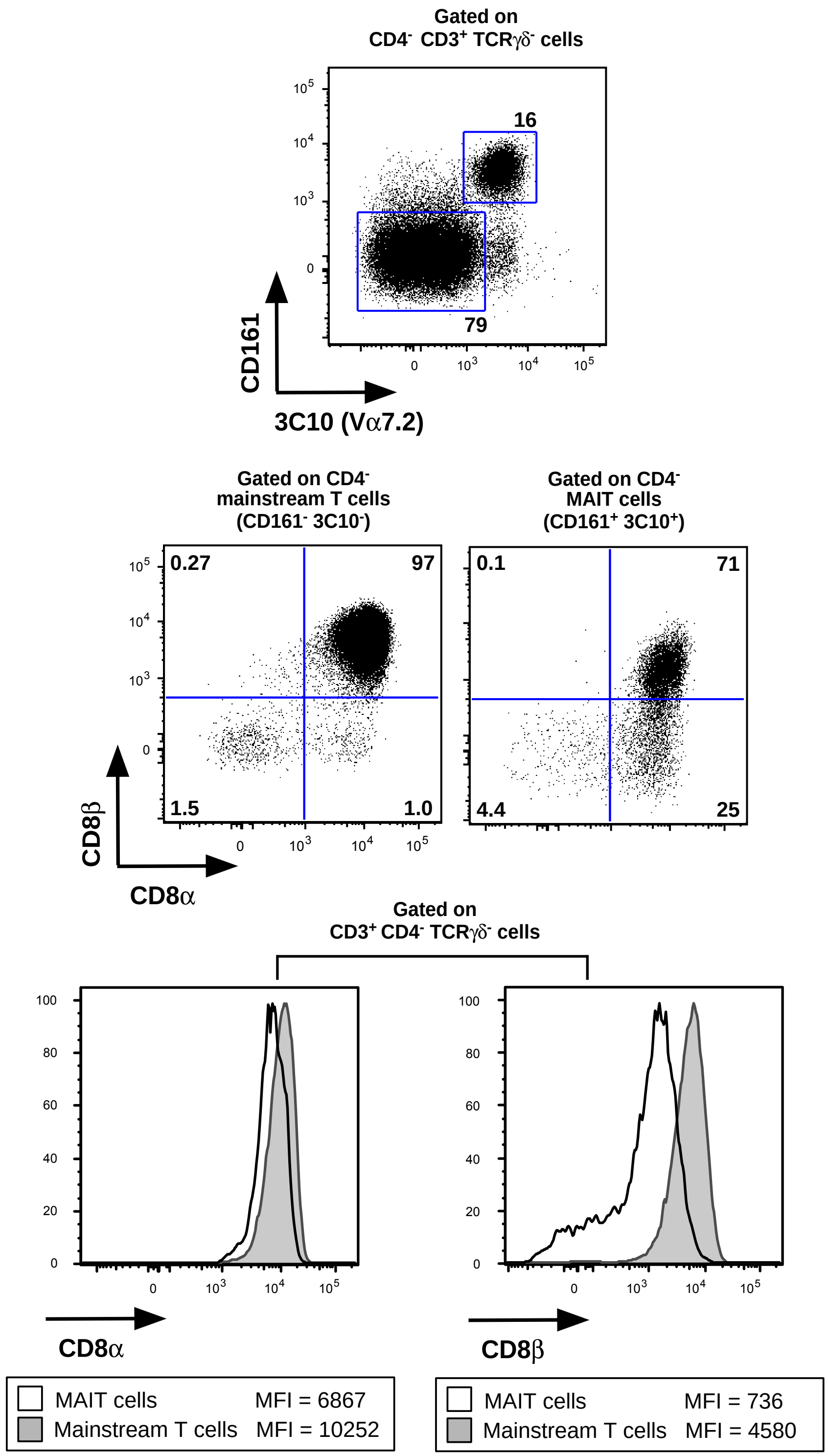
Gated on
CD4- CD3+ TCRγδ- cells
16
79
CD161
3C10 (Vα7.2)
Gated on CD4-
mainstream T cells
(CD161- 3C10-)
Gated on CD4-
MAIT cells
(CD161+ 3C10+)
0.27
97
1.5
1.0
0.1
71
4.4
25
CD8β
CD8α
Gated on
CD3+ CD4- TCRγδ- cells
CD8α
CD8β
MAIT cells MFI = 6867
Mainstream T cells MFI = 10252
MAIT cells MFI = 736
Mainstream T cells MFI = 4580
