# Supplementary material for: Stepwise Development of MAIT Cells in Mouse and Human
Source: PLoS Biol. 2009 Mar 10;7(3):e1000054. doi: 10.1371/journal.pbio.1000054 (PMC2653554; doi:10.1371/journal.pbio.1000054)
Supplement: Supplementary file 3 [file pbio.1000054.sg003.pdf]

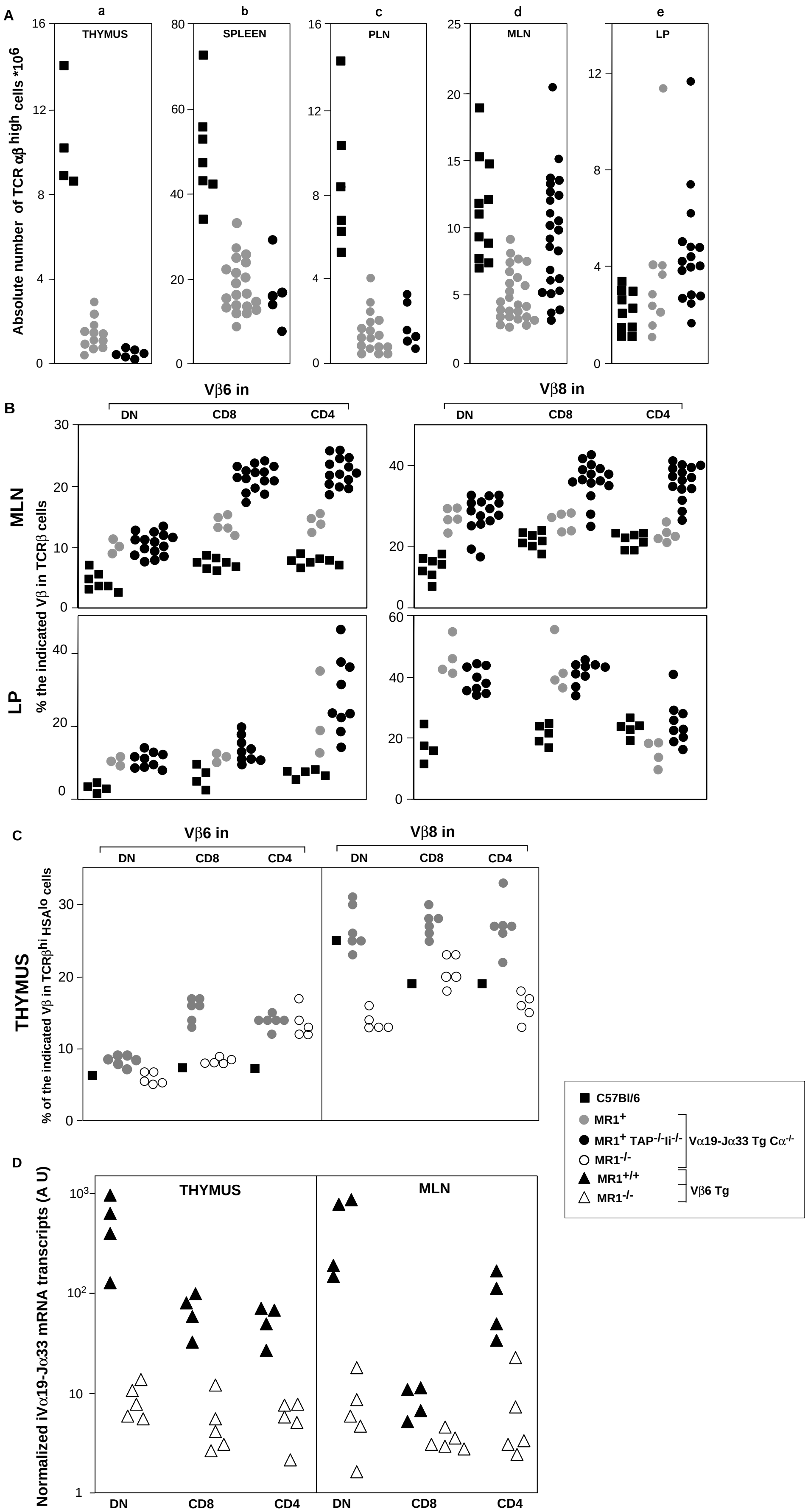
A
a
THYMUS
b
SPLEEN
c
PLN
d
MLN
e
LP
Absolute number of TCR αβ high cells *10^6
B
Vβ6 in
Vβ8 in
DN
CD8
CD4
MLN
LP
% the indicated Vβ in TCRβ cells
C
THYMUS
% of the indicated Vβ in TCRβhi HSAlo cells
D
THYMUS
MLN
Normalized iVα19-Jα33 mRNA transcripts (A U)
C57Bl/6
MR1+
MR1+ TAP-/-Ii-/-
MR1-/-
Vα19-Jα33 Tg Cα-/-
MR1+/+
MR1-/-
Vβ6 Tg
