# Supplementary material for: Stepwise Development of MAIT Cells in Mouse and Human
Source: PLoS Biol. 2009 Mar 10;7(3):e1000054. doi: 10.1371/journal.pbio.1000054 (PMC2653554; doi:10.1371/journal.pbio.1000054)
Supplement: Supplementary file 4 [file pbio.1000054.sg004.pdf]

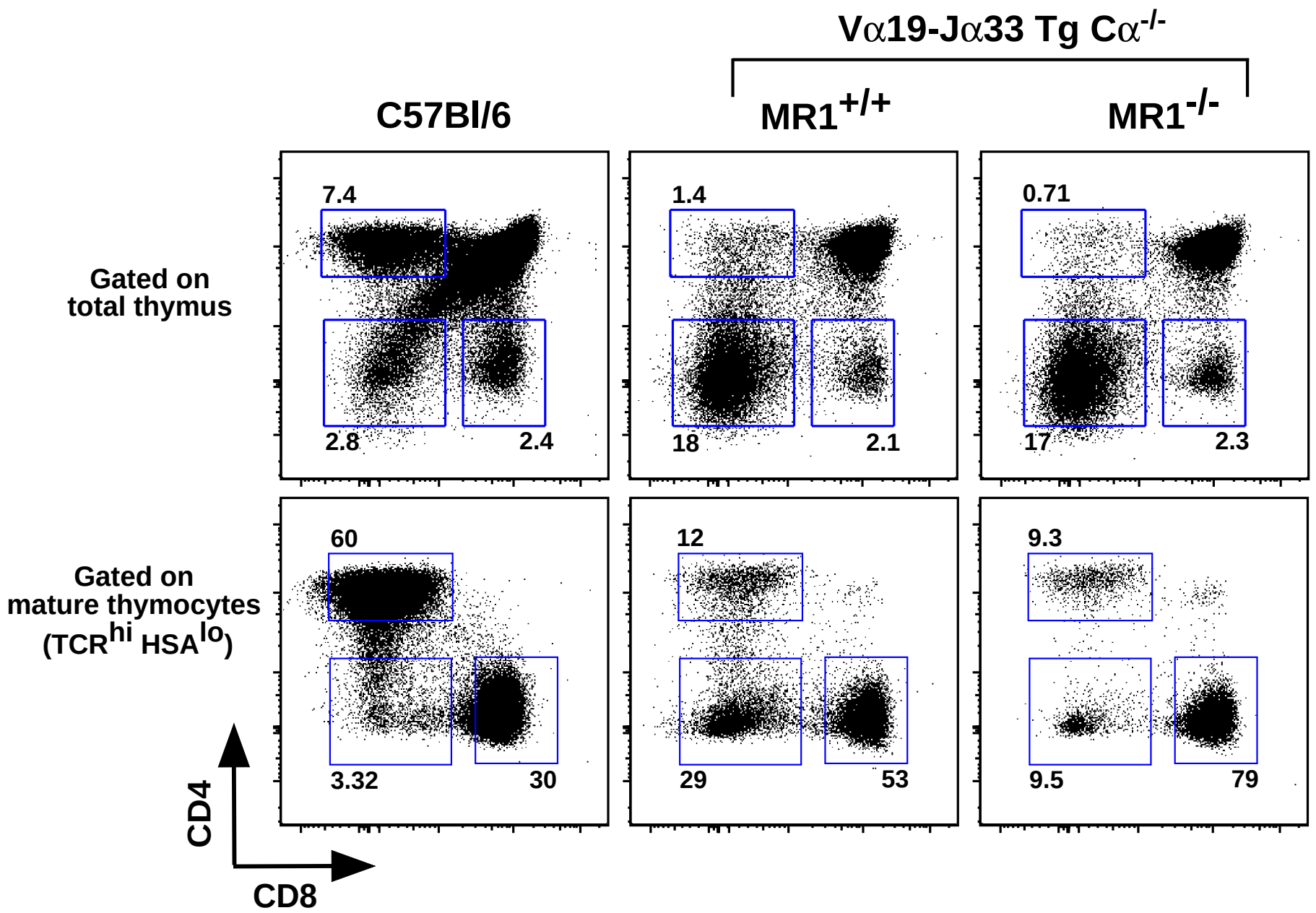
Vα19-Jα33 Tg Cα-/-
C57Bl/6
MR1+/+
MR1-/-
Gated on total thymus
7.4
2.8
2.4
1.4
18
2.1
0.71
17
2.3
Gated on mature thymocytes (TCRhi HSAlo)
60
3.32
30
12
29
53
9.3
9.5
79
CD4
CD8
