# Supplementary material for: Stepwise Development of MAIT Cells in Mouse and Human
Source: PLoS Biol. 2009 Mar 10;7(3):e1000054. doi: 10.1371/journal.pbio.1000054 (PMC2653554; doi:10.1371/journal.pbio.1000054)
Supplement: Supplementary file 5 [file pbio.1000054.sg005.pdf]

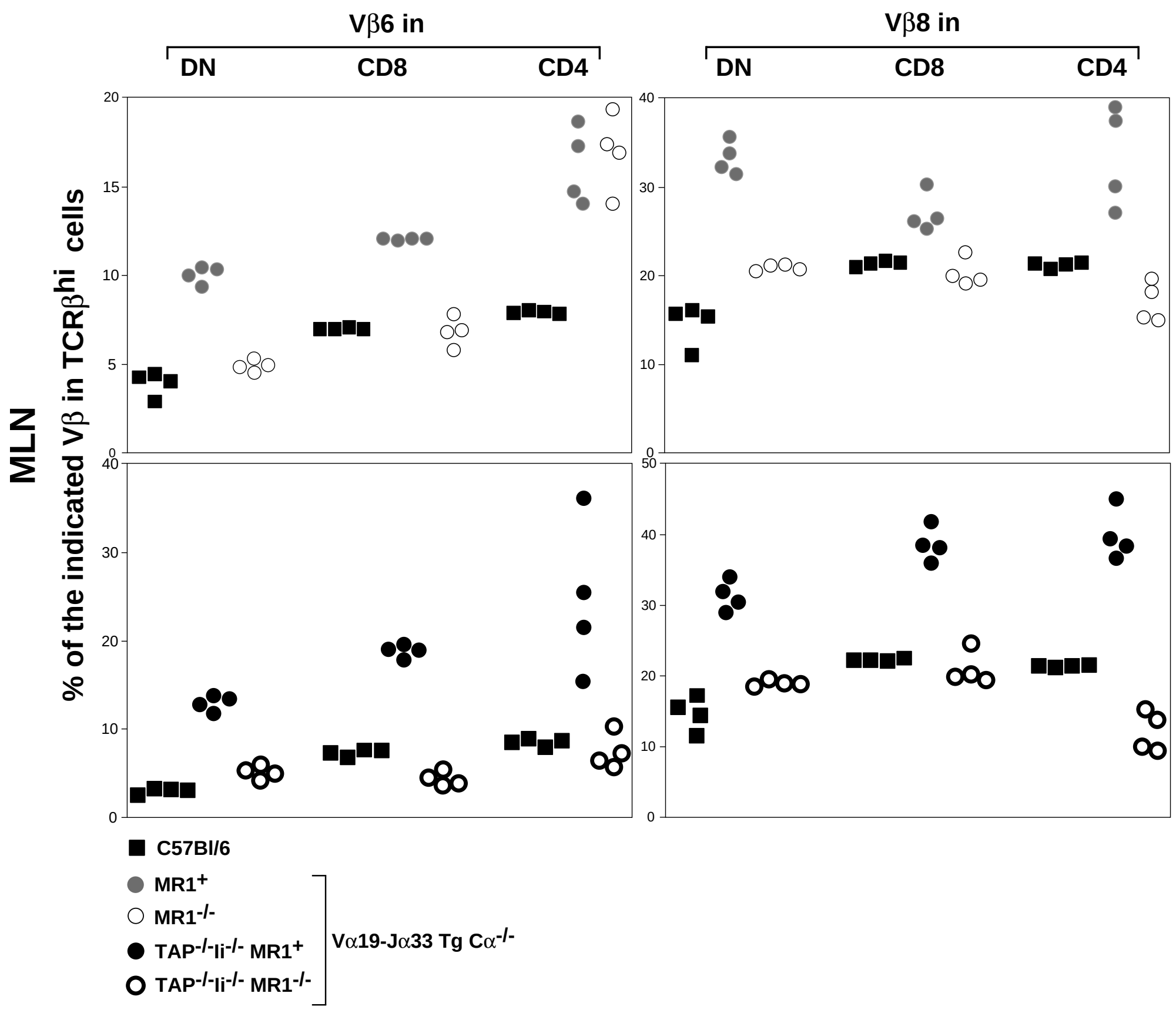

Vβ6 in
Vβ8 in
DN
CD8
CD4
MLN
% of the indicated Vβ in TCRβhi cells
C57Bl/6
MR1+
MR1-/-
TAP-/-Ii-/- MR1+
TAP-/-Ii-/- MR1-/-
Vα19-Jα33 Tg Cα-/-
