# Supplementary material for: Stepwise Development of MAIT Cells in Mouse and Human
Source: PLoS Biol. 2009 Mar 10;7(3):e1000054. doi: 10.1371/journal.pbio.1000054 (PMC2653554; doi:10.1371/journal.pbio.1000054)
Supplement: Supplementary file 6 [file pbio.1000054.sg006.pdf]

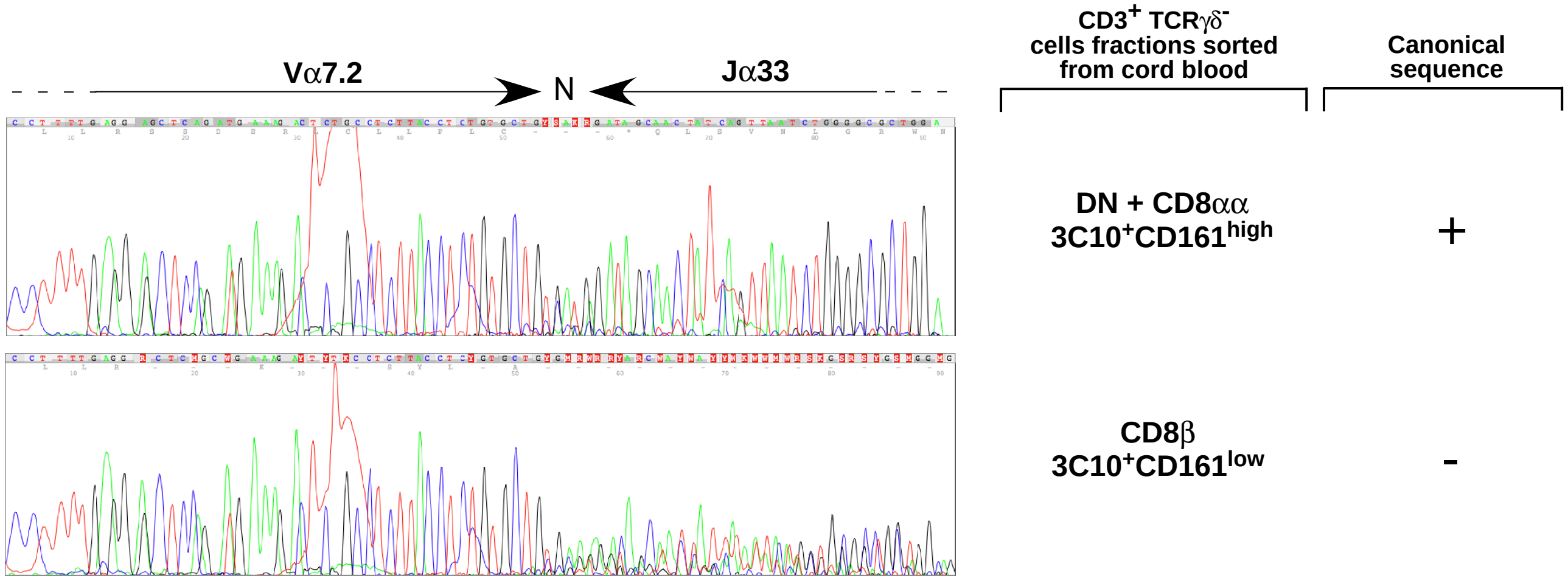
Vα7.2
N
Jα33
CD3+ TCRγδ-
cells fractions sorted
from cord blood
Canonical
sequence
DN + CD8αα
3C10+CD161high
+
CD8β
3C10+CD161low
-
