# Supplementary material for: Stepwise Development of MAIT Cells in Mouse and Human
Source: PLoS Biol. 2009 Mar 10;7(3):e1000054. doi: 10.1371/journal.pbio.1000054 (PMC2653554; doi:10.1371/journal.pbio.1000054)
Supplement: Supplementary file 7 [file pbio.1000054.sg007.pdf]

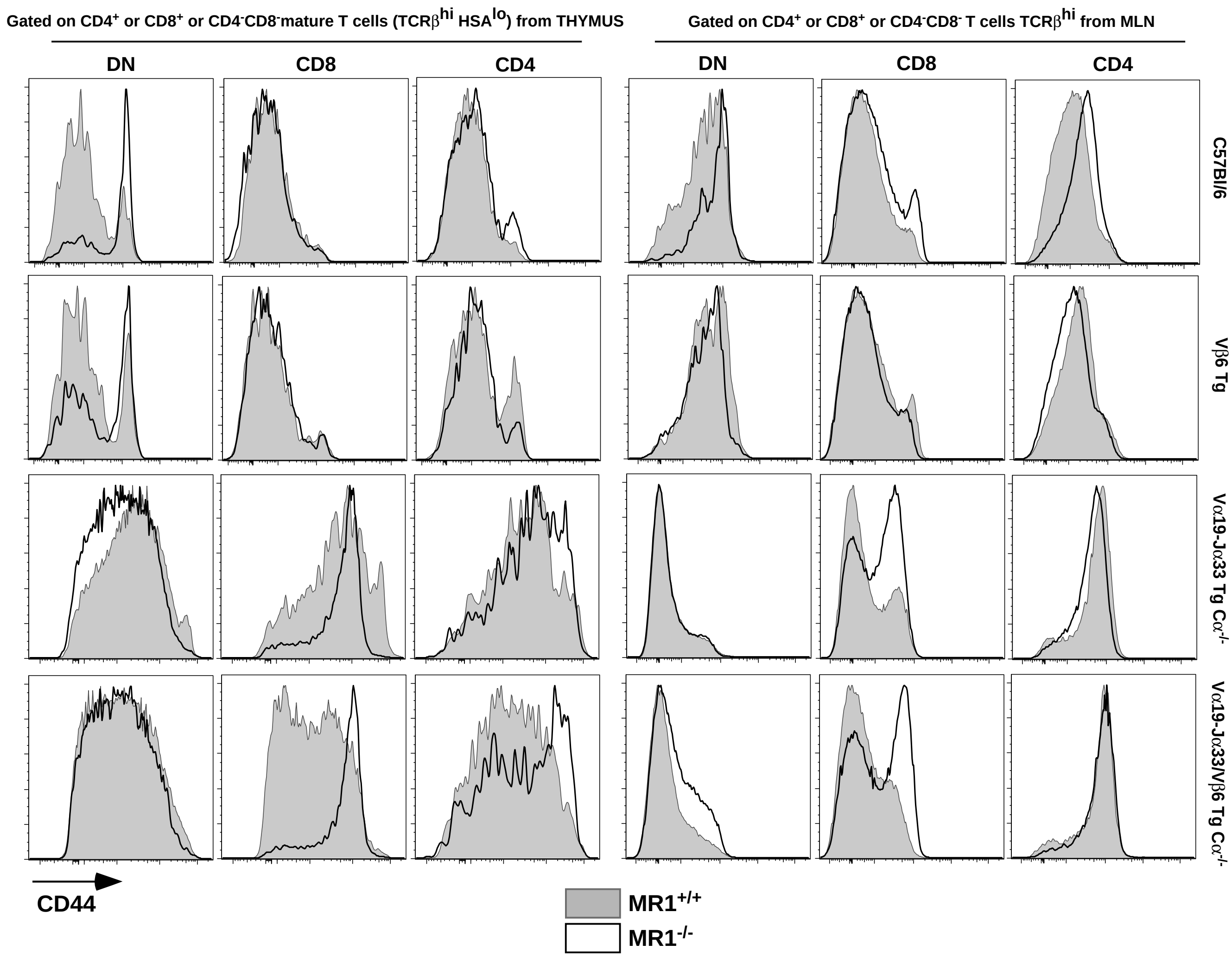
Gated on CD4+ or CD8+ or CD4-CD8- mature T cells (TCRβhi HSAlo) from THYMUS
Gated on CD4+ or CD8+ or CD4-CD8- T cells TCRβhi from MLN
DN
CD8
CD4
DN
CD8
CD4
C57Bl/6
Vβ6 Tg
Vα19-Jα33 Tg Cα-/-
Vα19-Jα33/Vβ6 Tg Cα-/-
CD44
MR1+/+
MR1-/-
