# Supplementary material for: Stepwise Development of MAIT Cells in Mouse and Human
Source: PLoS Biol. 2009 Mar 10;7(3):e1000054. doi: 10.1371/journal.pbio.1000054 (PMC2653554; doi:10.1371/journal.pbio.1000054)
Supplement: Supplementary file 8 [file pbio.1000054.sg008.pdf]

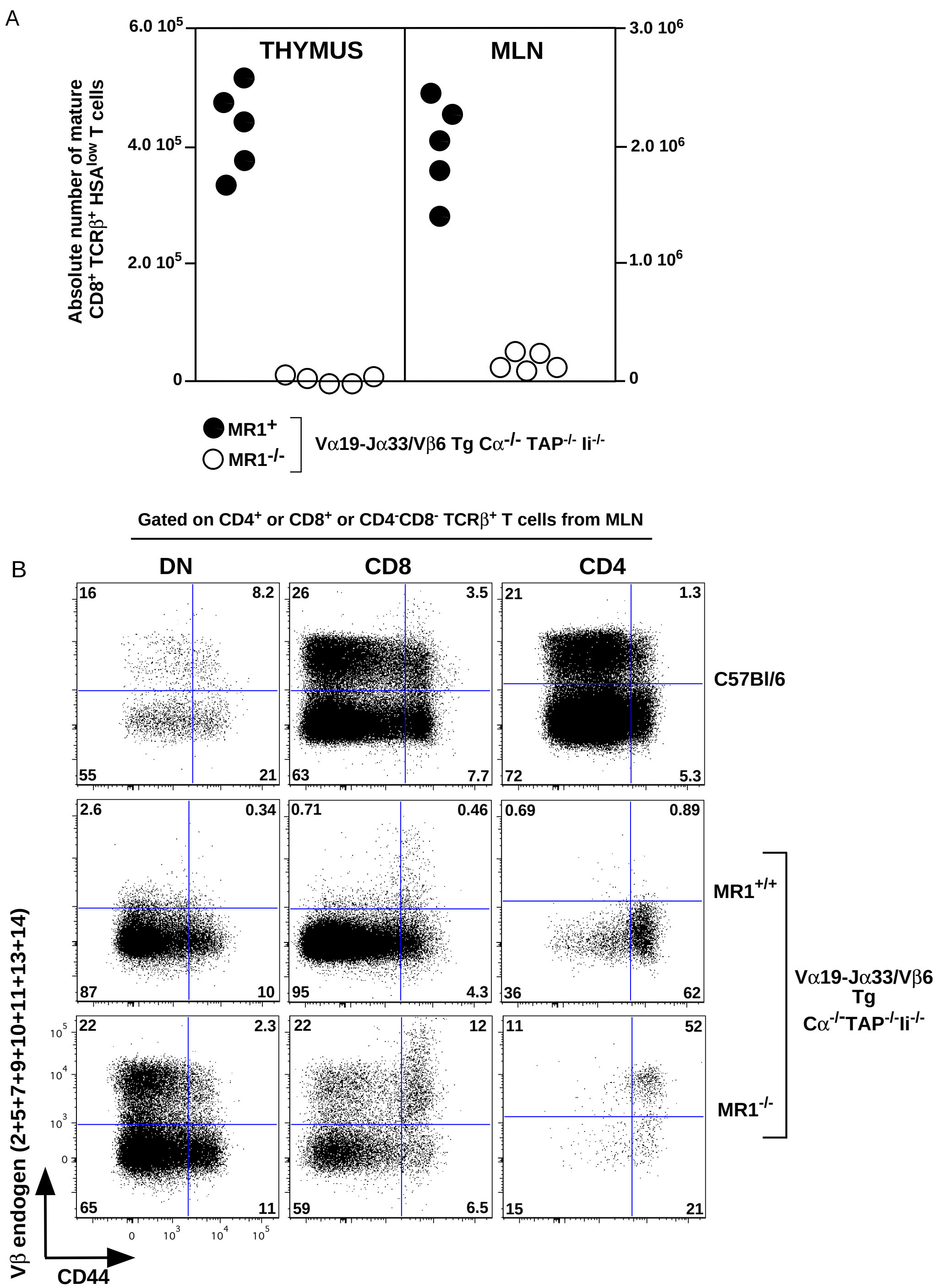

A
THYMUS
MLN
Absolute number of mature CD8+ TCRβ+ HSAlow T cells
6.0 10^5
4.0 10^5
2.0 10^5
0
3.0 10^6
2.0 10^6
1.0 10^6
0
MR1+
MR1-/-
Vα19-Jα33/Vβ6 Tg Cα-/- TAP-/- Ii-/-
Gated on CD4+ or CD8+ or CD4-CD8- TCRβ+ T cells from MLN
B
DN
CD8
CD4
C57Bl/6
MR1+/+
MR1-/-
Vα19-Jα33/Vβ6 Tg Cα-/-TAP-/-Ii-/-
Vβ endogen (2+5+7+9+10+11+13+14)
CD44
16
8.2
55
21
26
3.5
63
7.7
21
1.3
72
5.3
2.6
0.34
87
10
0.71
0.46
95
4.3
0.69
0.89
36
62
22
2.3
65
11
22
12
59
6.5
11
52
15
21
