# Supplementary material for: Stepwise Development of MAIT Cells in Mouse and Human
Source: PLoS Biol. 2009 Mar 10;7(3):e1000054. doi: 10.1371/journal.pbio.1000054 (PMC2653554; doi:10.1371/journal.pbio.1000054)
Supplement: Supplementary file 9 [file pbio.1000054.sd001.doc]

**Text S1, Martin et al.: Description and discussion of the iVα19 TCRα chain and Vβ6 TCRβ chain single transgenic mice.**

*Generation of mice over-expressing MAIT cells: mice transgenic for the canonical iVa19 TCRα chain*

Three different founder lines were analyzed and backcrossed onto a Cα^-/-^ B6 background to prevent the expression of endogenous TCRα chains. However, Cα^-/-^ iTCRα chain Tg mice may potentially rearrange any TCRβ chain and generate a very diverse TCR repertoire with or without MR1 restriction. Given the lack of known MAIT cells-specific marker(s), this would make it impossible to distinguish mainstream MHC class Ia- and class II-restricted T cells from MR1-specific MAIT cells. As MAIT cell selection is independent of both transporter associated protein (TAP) [1] and invariant chain (Ii) expression [2], we also crossed the mVα19-Jα33 Tg lines onto a triple KO Cα^-/-^/TAP^-/-^/Ii^-/-^ background, to decrease the expression of MHC class Ia and II molecules, thereby eliminating most mainstream T cells. As MAIT cells express the Vβ6- and Vβ8- TCRβ chains in large excess [1], we focused our analysis on these segments, which should be over-represented in our Tg mice. Finally, we took into account the "transgenic" artifacts generated by the expression of a rearranged TCRα chain early in T-cell ontogeny [3,4], by always comparing the phenotype and characteristics of T cells from MRI^+^ and MR1^neg^ mice. Only features present in MR1^+^ but not in MR1-deficient TCR Tg mice were considered as MAIT cell specific.

*T cell phenotype of iVα19-Jα33 Tg mice*

A preliminary analysis of the iVα19 Tg lines showed that the level of TCRβ chain expression varied slightly according to the founder, but that the numbers of lymphocytes in the different organs and CD4/CD8 subset proportions were similar in different founders (not shown). We therefore display pooled data from the various founders. iVα19 Tg mice had far fewer TCRαβ^hi^ thymocytes than wt animals (1.1x10^6^, 0.21x10^6^ and 10.2x10^6^ for iVα19 Tg mice on a Cα^-/-^ or Cα^-/-^/TAP^-/-^/Ii^-/-^ background and in B6 mice, respectively) (Suppl. Fig. 3Aa). Tg mice also had fewer T cells in the spleen (Suppl. Fig. 3Ab) and peripheral lymph nodes (PLN) (Suppl. Fig. 3Ac). By contrast, the number of Tg T cells in the mesenteric lymph nodes (MLN) was about 50% that in controls and reached or even exceeded control levels in the Lamina Propria (LP) (suppl. Fig. 3Ad, e). The low frequency of Tg T cells in the thymus, spleen and PLN and the accumulation of these cells in the MLN and gut LP was even more obvious in the absence of classical MHC molecules (Cα^-/-^TAP^-/-^Ii^-/-^) than in their presence (Cα^-/-^). Thus, the numbers of T cells in the different organs of iVα19 Tg mice parallel the tissue distribution of MAIT cells observed in wt animals [1,2].

We found that iVα19 Tg mice had a larger numbers of DN T cells in the MLN and LPL than wt mice, with fewer CD4^+^ T cells in the MLN (Suppl. Fig. 4). Further validation of our iVα19 Tg mice was achieved by studying the Vβ6/8 repertoire of the T cells present in the different organs. In a Cα^-/-^ background, the level of Vβ6 usage was doubled in all CD4/CD8 subsets whereas Vβ8 usage was 1.5 times higher than in the wild type in both the spleen (data not shown) and MLN (Suppl. Fig. 3B). In the absence of classical MHC molecules (Cα^-/-^TAP^-/-^Ii^-/-^ background), iVα19 Tg T cells displayed an even more profound bias in Vβ repertoire, as Vβ6+8 positive T cells accounted for about 60 % of all T cells, versus less than 30 % in wt mice (Suppl. Fig. 3B and data not shown). Thus, transgenic over-expression of the invariant TCRα chain induces a clear bias in Vβ chain segment usage towards Vβ6 and Vβ8, reproducing the MAIT cell repertoire of our iVα19 T-T hybridoma [1].

Comparison of Vβ6/Vβ8 bias in the presence or absence of MR1 provided us with an estimate of T cell selection by MR1. In the absence of classical MHC molecules (iVα19 Tg Cα^-/-^TAP^-/-^Ii^-/-^ mice), the Vβ6/Vβ8 bias disappeared in the absence of MR1 in DN and CD8 T cells, in both the MLN and LP (Suppl. Fig. 5 and data not shown). The observed differences in Vβ6/Vβ8 segment usage between MR1^+^ and MR1-deficient backgrounds indicate that at least 50 % of the T cells are MR1 restricted. In the presence of classical MHC molecules, the smaller Vβ6/8 bias also disappeared in the absence of MR1 (Suppl. Fig. 5). Thus, iVα19 Tg mice select a large number of MR1-restricted T cells, which display MAIT cell features in the periphery, in terms of tissue location and Vβ repertoire usage.

In addition, the Vβ6/8 bias varied according to co-receptor usage and the tissue analyzed: in the MLN, both the Vb6 and Vb8 bias was more apparent in the CD4 and CD8 subsets, whereas in the LP, strong Vβ6 expression was restricted to the CD4 subset and Vβ8 was used mostly by DN and CD8 T cells (Suppl. Fig 3B). These results suggest that subtle differences in the putative ligands presented by MR1 [5] in the LP and MLN may lead to differential expansion of the MAIT cells, according to their fine specificity.

The MR1-dependent increase of Vβ6/Vβ8^+^ MAIT cells observed in iVα19 Tg mice made it possible to investigate whether these cells were present in the thymus. In the thymus of these mice, there was a clear increase in DN and CD8 subsets (Suppl. Fig. 4). In the presence of classical MHC molecules, the CD4, CD8 and DN subsets of mature thymocytes displayed higher levels of Vβ8 segment usage in MR1-positive mice than in MR1-deficient mice (Suppl. Fig. 3C). An MR1-dependent increase in Vβ6 usage was also found in the CD8 and, to a lesser extent, DN subset. However, in the CD4 thymocytes, as well as in the MLN CD4 T cells (Suppl*.* Fig 3C and Suppl. Fig. 5 upper panel), the Vβ6 bias did not disappear in the absence of MR1*,* suggesting that these cells were restricted by other MHC molecules. Indeed, the number of Vβ6/CD4 T cells decreased to B6 level in the absence of either Ii chain (data not shown) or both Ii and TAP (Suppl. Fig. 5), showing that a small subset of iVα19/Vβ6^+^ CD4^+^ is probably selected on a classical MHC class II molecules, at least in these transgenic animals. In line with these data, in the absence of classical MHC molecules, the small number of mature thymocytes found in iVα19 Tg Cα^-/-^TAP^-/-^Ii^-/-^ mice decreased even further and only a few mature DN and almost no SP remained in the absence of MR1 (data not shown). Thus, MAIT cells are present in the thymus of iVα19 Tg mice, resulting in an MR1-dependent increase in the percentage of mature thymocytes expressing Vβ6 and Vβ8.

##### MR1-dependent increase in the frequency of iVα19^+^ T cells in mice transgenic for a MAIT cell Vβ6 TCRβ chain

Two founder lines were studied in a B6 background. The distribution of CD4, CD8 and DN thymocytes were only slightly modified by Vβ6 Tg expression (not shown). We assessed the frequency of MAIT cells in the different subsets by FACS-mediated sorting of CD4, CD8 and DN TCRβ^+^ lymphocytes from the MLN of Vβ6 TCRβ Tg mice in MR1^+^ and MR1-deficient background followed by quantification of iVα19-Jα33 transcripts. MR1-proficient mice had 100 times as many iVα19 transcripts as MR1-deficient mice in DN cells, and 10 times as many such transcripts in CD4 T cells, whereas no such difference was observed in CD8 T cells. Similarly, the thymus of MR1-positive mice contained larger numbers of iVα19 transcripts in the DN (100 times as many) and CD4 (10 times), but also in CD8 (10 times) mature thymocytes, than observed in the absence of MR1 (Suppl. Fig. 3D). The dependence of invariant TCRα transcript levels on MR1 expression indicates that the iVα19 T cells observed in the Tg mice were bona fide MR1-restricted MAIT cells. Thus, Vβ6 Tg mice, which are less prone to artifacts than TCRα Tg mice, select a high number of MR1-restricted T cells, which are readily detectable in the thymus, confirming the results obtained in iVα19 Tg mice.

1. Tilloy F, Treiner E, Park SH, Garcia C, Lemonnier F, et al. (1999) An invariant T cell receptor alpha chain defines a novel TAP-independent major histocompatibility complex class Ib-restricted alpha/beta T cell subpopulation in mammals. J Exp Med 189: 1907-1921.

2. Treiner E, Duban L, Bahram S, Radosavljevic M, Wanner V, et al. (2003) Selection of evolutionarily conserved mucosal-associated invariant T cells by MR1. Nature 422: 164-169.

3. Terrence K, Pavlovich CP, Matechak EO, Fowlkes BJ (2000) Premature expression of T cell receptor (TCR)alphabeta suppresses TCRgammadelta gene rearrangement but permits development of gammadelta lineage T cells. J Exp Med 192: 537-548.

4. Erman B, Feigenbaum L, Coligan JE, Singer A (2002) Early TCRalpha expression generates TCRalphagamma complexes that signal the DN-to-DP transition and impair development. Nat Immunol 3: 564-569.

5. Huang S, Gilfillan S, Cella M, Miley MJ, Lantz O, et al. (2005) Evidence for MR1 antigen presentation to mucosal-associated invariant T cells. J Biol Chem 280: 21183-21193.
